# Time-course transcriptome and WGCNA analysis revealed the drought response mechanism of two sunflower inbred lines

**DOI:** 10.1371/journal.pone.0265447

**Published:** 2022-04-01

**Authors:** Yang Wu, Yaru Wang, Huimin Shi, Haibo Hu, Liuxi Yi, Jianhua Hou

**Affiliations:** Agricultural College, College of Agricultural, Inner Mongolia Agricultural University, Hohhot, China; ICAR-National Institute of Plant Biotechnology, New Delhi, INDIA

## Abstract

Drought is one of the most serious abiotic stress factors limiting crop yields. Although sunflower is considered a moderate drought-tolerant plant, drought stress still has a negative impact on sunflower yield as cultivation expands into arid regions. The extent of drought stress is varieties and time-dependent, however, the molecular response mechanisms of drought tolerance in sunflower with different varieties are still unclear. Here, we performed comparative physiological and transcriptome analyses on two sunflower inbred lines with different drought tolerance at the seedling stage. The analysis of nine physiological and biochemical indicators showed that the leaf surface area, leaf relative water content, and cell membrane integrity of drought tolerance inbred line were higher than those of drought-sensitive inbred line under drought stress, indicating that DT had stronger drought resistance. Transcriptome analyses identified 24,234 differentially expressed genes (DEGs). Gene ontology (GO) analysis showed the up-regulated genes were mainly enriched in gibberellin metabolism and rRNA processing, while the down-regulated genes were mainly enriched in cell-wall, photosynthesis, and terpene metabolism. Kyoto Encyclopedia of Genes and Genomes(KEGG) pathway analysis showed genes related to GABAergic synapse, ribosome biogenesis were up-regulated, while genes related with amino sugar and nucleotide sugar metabolism, starch and sucrose metabolism, photosynthesis were down-regulated. Mapman analysis revealed differences in plant hormone-signaling genes over time and between samples. A total of 1,311 unique putative transcription factors (TFs) were identified from all DEGs by iTAK, among which the high abundance of transcription factor families include bHLH, AP2/ERF, MYB, C2H2, etc. Weighted gene co-expression network analysis (WGCNA) revealed a total of 2,251 genes belonging to two modules(blue 4, lightslateblue), respectively, which were significantly associated with six traits. GO and KEGG enrichment analysis of these genes was performed, followed by visualization with Cytoscape software, and the top 20 Hub genes were screened using the CytoHubba plugin.

## Introduction

Sunflower(*Helianthus annuus L*.) is a globally important oilseed food that is mainly cultivated for the production of edible oil, with the seeds containing 36–52% edible oil and 28–32% protein [1]. It is considered with moderately drought resistant since it has a strong root system that can absorb water from deeper soils [2,3], and with high photosynthetic potential due to the presence of stomata on both sides of the leaf [4]. However, since it grows mostly in tropical and subtropical regions, it is more susceptible to drought, which causes seed and oil yield to decline [5]. The effect of drought on sunflowers is multi-level, with corresponding changes from phenotypic to physiological and biochemical indicators, such as decreased plant height, leaf surface area (LSA), leaf relative water content(RWC) [6,7], increased root length, and the root-shoot ratio [8], closed stomata [6], decreased photosynthesis level [6], shrinkage in cell volume [9], reduced water potential [4] and membrane stability [10], disrupted the balance of reactive oxygen [11]. The extent of the drought effects depends on plant genotype, stress intensity, crop growth period, and stress duration [12,13]. Obviously, the best way to alleviate drought stress is to irrigate fields, but this is not achievable for some farmers due to the increased costs, labor inputs, and lack of water resources or/and infrastructure [14]. Since the morphological and physiological alteration of plants induced by the breeding method is heritable, genetic engineering to breed drought-resistant varieties is probably the most successful and cheapest strategy to combat drought [15]. Drought tolerance is a complex quantitative trait controlled by many genes [16], one of the most difficult issues in sunflower genetic breeding is to find the genes that really affect drought tolerance among the huge number of genes. Fortunately, with the publication of sunflower genome sequence [17] and the development of next-generation sequencing technology, transcriptome analysis provides a powerful weapon for insight into the cellular and molecular responses to drought stress in sunflower [18].

Several studies have been conducted on different varieties of sunflower under drought stress have provided a basis for drought-resistant sunflower genetic breeding [19–21].

S Moschen et al. [22] used a sunflower hybrid to carry out drought stress in three development stages(seedling, pre-flowering, post-flowering). Through the joint analysis of transcriptome and metabolomics, they gave some new and important insights on the drought-resistant response mechanism of sunflower. Liang et al. [23] conducted transcriptome analysis of sunflower roots and leaves under polyethylene glycol simulated drought conditions for 24 hours, and found the tissue specificity of gene expression, in which more differentially expressed genes (DEGs) was obtained in leaves. All of these studies used only one genotype of sunflower. According to the research of Sarazin Vivien et al. [24], there are genotypic differences in water stress response, so we can have a deeper understanding of the drought-resistant response mechanism of sunflower by comparing the phenotypes and transcriptome differences of different varieties under the same condition.

In this study, we used two sunflower inbred lines with a significant difference in drought resistance and conducted pot drought stress experiments at the seedling stage. We then measured 9 physiological and biochemical indexes under drought stress and normal water conditions respectively. A large number of DEGs were identified, functional annotation and enrichment analysis on these genes was conducted. As a supplement, the weighted gene co-expression network analysis (WGCNA) R package [25] clusters the DEGs by their expression level among different samples, and then carries out correlation analysis to find the gene sets directly related to a certain trait, thus improving the applicability of RNA-seq results. By using WGCNA, we identified two modules related to six traits, and made further analysis on the genes in these modules. This is the first study of drought stress in sunflowers using this combined analysis method, and our results provide a basis for future selective gene research and molecular marker-assisted breeding.

## Materials and methods

### Plant materials and growth conditions

The research was conducted in the city of Hohhot (111.71, 40.819, 1000 m above the sea level), Inner Mongolia Province, China. In early August 2020. The drought-sensitive inbred lines K55(denoted as ‘DS’) and the drought-tolerant K58(denoted as ‘DT) that selected from 226 varieties in our previous study. Seeds were sterilized with 3% sodium hypochlorite for 5 min, followed by washing 3 times with sterile distilled water. Then germinated on two layers of wet filter papers in petri-dishes at room temperature (19°C to 25°C) for 24 h according to the procedures of Lei et al [26]. Germinated seeds were sowed in PVC cultivate pots (25 cm×19 cm×16 cm) with 3.5kg uniform soil (containing vermiculite, nutritional soil, field soil (1:1:1)), and grown under greenhouse (light/dark cycles: 14/10; 28/22°C; 45±5% relative humidity) without water and nutritional limitations. A total of six PVC pots were used in this study. Five uniformed seeds were planted in each pot as one replicate with three biological replicates per variety.

### Drought treatment and sampling

After the third leaf was fully expanded, all seedlings were stopped irrigation and subjected to drought stress according to Pereyra et al [27]. Based on our pre-experiment, the water content of about 10% can trigger sunflower drought response, and keep them alive during the experiment, so we controlled the soil water content at 8±3%, and recorded the day when the soil water content first dropped to 30% as 0-day, then the seedlings of both varieties were subjected to drought stress for 0 to 21 days. The soil water content of each pot was determined at 9 a.m. every morning using the gravimetric method described by Martin Enrique Tagliotti et. al [28], and replenished water according to the target soil water content. Sampling was performed after 0, 7, 14, and 21 days of drought treatment. Six fully expanded young leaves were randomly excised from each pot for the determination of physiological/biochemical indicators and RNA extraction, and the leaves used for RNA extraction were immediately frozen in liquid nitrogen and stored at −80°C till the RNA isolation.

### Determination of physiological and biochemical indexes

A total of nine physiological and biochemical traits were measured in this study. LSA was calculated using the length-width coefficient method [29]. The relative electric conductivity(REC) was measured using the electrical conductivity method described by Lutts et al [30]. RWC was determined using the saturate water method [31]. Leaf superoxide dismutase(SOD) activity was measured based on its ability to inhibit the photochemical reduction of nitro blue tetrazolium at 560 nm [32]. Catalase activity(CAT) was determined by measuring H_2_O_2_ consumption at 240 nm [33]. The leaf peroxidase (POD) activity was estimated by the guaiacol colorimetric assay by monitoring the changes of absorption at 470 nm accompanied by guaiacol oxidation [34]. The free proline content (Pro) was measured using a spectrophotometer according to the method of Bates et al [35]. The Soluble Sugar(SS) was measured according to the procedure described by Dubois [36]. The level of malondialdehyde(MDA) content was determined by a modified thiobarbituric acid chromatometry method [37].

### RNA extraction and cDNA library construction and sequencing

Total mRNA was isolated using the RNA prep Pure Plant Kit DP411(Tiangen, Tianjin, China) according to the instruction manual. RNA concentration and purity were measured using a NanoDrop 2000 spectrophotometer(Thermo Fisher Scientific, Wilmington, DE). RNA integrity was assessed using an Agilent 2100 LabChip GX (Agilent Technologies, CA, USA) by 1% agarose gel electrophoresis. A single library requires 2μg of RNA, with a concentration ≥ 40 ng/μL, OD260/280 between 1.7–2.5, OD260/230 between 0. 5–2. 5, and RNA integrity number (RIN) > 6.5. The mRNA was then enriched with Oligo (dT) magnetic beads. The mRNA was added with fragmentation buffer and cut into short fragments. RNA was reverse transcribed into cDNA using six-base random primers. The double-stranded cDNA samples were purified, end-repaired, added with poly(A) tails, and then ligated to the sequencing adapters to create cDNA libraries. After the libraries had passed the quality test, all samples were sequenced from both ends with a Novaseq 6000 (Illumina) system and sequences of paired 150-bp were obtained.

### Read mapping and differential expression analysis

Raw reads of sequencing were processed by FastQC (version 0.11.9) for quality control. Meanwhile, Q30, GC-content, and sequence duplication levels were calculated. High-quality clean reads were mapped to the sunflower reference genome(GeneBank NO. GCA_002127325. 2) [17] using Hisat2 (version 2.2.1) software [38] with default parameters.

The alignment SAM files were converted to BAM format using Samtools (version 1.11), The processed BAM files were further processed with StringTie software(2.1.4) [39]. Expression levels of the genes were calculated using fragments per kilobase per million reads(FPKM). The correlation heatmap and principal component analysis (PCA) analysis were performed using the Corrplot R packages (0.89). Normalization and DEGs analysis were performed using DESeq2 [40]. The samples were divided into six groups (DS7 vs. DS0, DS14 vs. DS0, DS21 vs. DS0, DT7 vs. DT0, DT14 vs. DT0, DT21 vs. DT0). Genes with padj≤0. 01 and |log_2_(Fold Changes)| ≥ 1 was considered to be DEGs.

### Functional annotation and enrichment analysis

Gene ontology(GO) and Kyoto Encyclopedia of Genes and Genomes(KEGG) analysis were performed to reveal the biological functions of genes and pathways of DEGs by ClusterProfiler(version 4.0.0) R-package [41]. Fasta files were input to Eggnog (version 2. 0. 1) [42] to obtain functional annotations. The annotation package was constructed using the AnnotationForge R package. REVIGO program(http://revigo.irb.hr/) was used to remove redundant GO-terms [43]. The iTAK software was used to identify and classify plant transcription factors(TFs) [44].

### WGCNA network analysis

WGCNA analysis [25] was conducted to construct co-expression networks of all DEGs. The cutreeStatic function was used to remove the offending sample. The soft thresholding power β was chosen based on the lowest power for which the scale-free topology fit index reached a high value. ExportNetworkToCytoscape function was used to exported network edge and node information of genes in each module, The Cytoscape (version 3.8.2) [45] was used to visualize the network. The plug-in cytoHubba was used to identify the top 20 hub genes with maximal clique centrality (MCC) computing method.

### Validation of the DEGs by RT-qPCR

To verify the RNA-seq results, we randomly selected 10 DEGs for Quantitative reverse transcription PCR(RT-qPCR) analysis. Total RNA of 24 samples was isolated from leaves by Trizol reagent (Shenggong, Beijing, China) following the protocol. The integrity of RNA was confirmed by agarose gel electrophoresis with 4S Green Plus (Shenggong, Beijing, China) staining of 5S, 18S, and 28S RNA. Samples that had an A260/280 ratio of 1.8–2.0 and A260/A230 ratio of 2–2.5 were considered acceptable, Reverse transcription was performed using standard procedures of the BiomarkerScript II 1st Strand cDNA Synthesis Kit (Biomarker). RT-qPCR analysis was performed in FTC-3000TM real-time quantitative thermal cycler (Funglyn Biotech, Toronto, Canada) using a Biomarker 2X SYBR Green Fast qPCR Mix (Biomarker, Beijing, China). Gene-specific primers were designed using a free online primer design tool (https://www.sangon.com/newPrimerDesign), and were synthesized by Shenggong Inc. The detail of primers was shown in the S1 Table. Three technical replicates were carried out to ensure the reproducibility of results. Genes expression levels were calculated from the threshold cycle according to 2^-△△Ct^ [46] and standard deviation was calculated between three biological replicates. 18S rRNA gene was used as the endogenous control [47].

### Statistical analysis

All physiological and biochemical indexes were statistically analyzed using IBM Student’s t-test of Statistical Package for the Social Sciences (IBM SPSS Statistics for Windows, Version 20.0. Armonk, NY: IBM Corp.) and all data were presented as the mean ± SD (n = 3). The data were analyzed by one-way analysis of variance (ANOVA). Means were compared by the Tukey’s honestly significant difference (Tukey’s HSD) method at the p<0. 05. The bar graphs were drawn by Graphpad Prism (v8.0.2).

## Results

### Physiological difference between DS and DT under drought-stress

**Fig 1:**
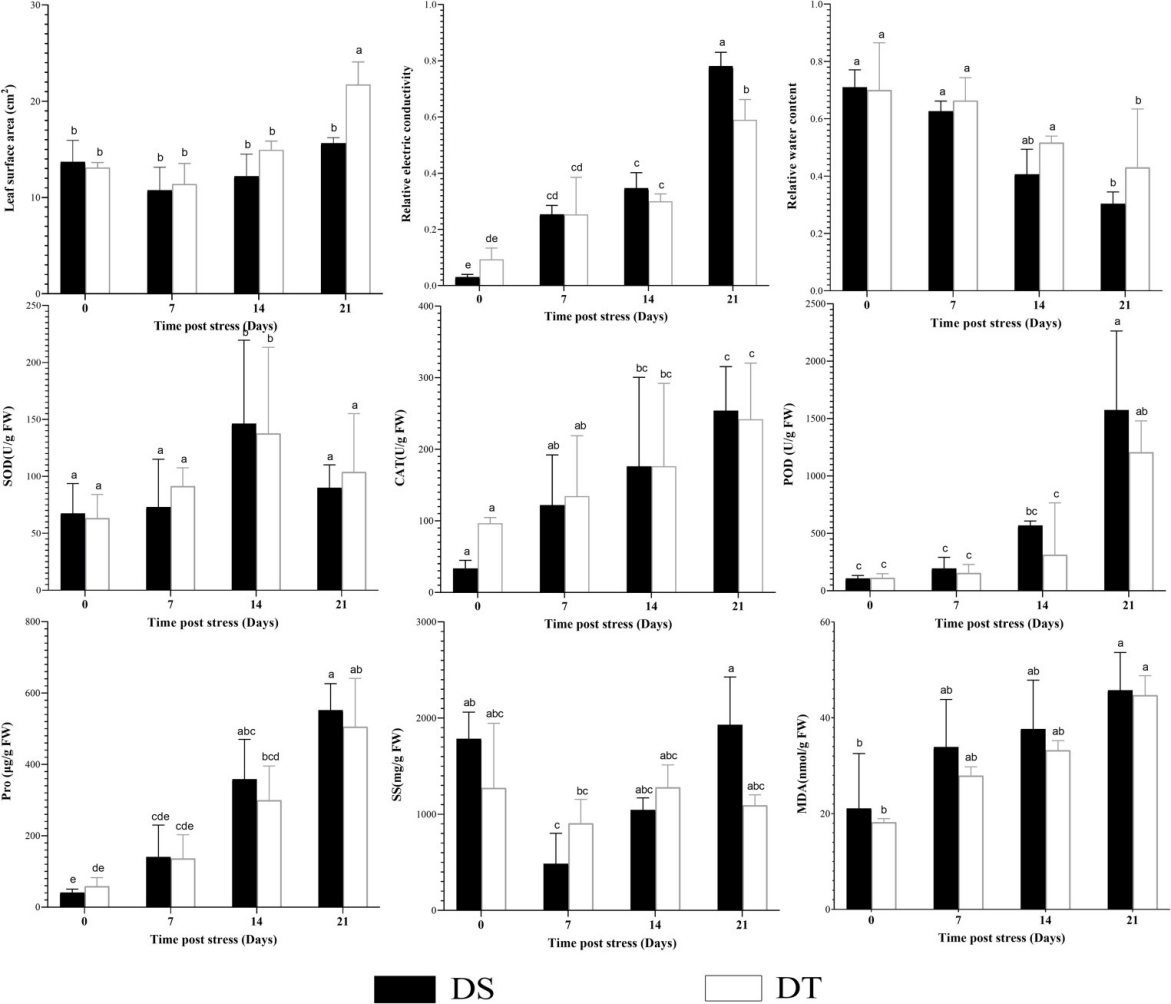
Physiological and biochemical traits of DS and DT under drought stress. Error bars denote standard error of the mean. Significant differences between samples at p≤0. 05 were denoted by different letters.

The LSA of both varieties decreased in the early stage (day 7) of drought stress, and a subsequent increase in the later stage, while the rate and magnitude of the increase were greater in the DT than in DS. Sustained drought stress resulted in a reduction in RWC, and DS decreased more rapidly than DT. The REC, CAT, POD, and Pro were increased with the drought stress time, while a bigger level of increase was observed in DS than in DT (Fig 1, S2 Table). A similar trend was observed for SOD during 0 to 14 day, but it dropped on the 21 day in both varieties. SS increased from 7 day to 14 day after a decrease at the early stage (0–7 day) in two varieties. Then there was an opposite trend at 21 day between DS and DT, the former decreasing and the latter increasing. MDA content continually increases with the duration of drought stress, but no significant difference was observed between the two varieties.

### RNA sequencing and mapping

A total of 193.33 Gb clean data were obtained after quality control. The Q30 value of each library ranged from 93.57% to 94.97%, and the GC content ranged from 44.82% to 45.85%. The mapping ratio ranged from 85.71% to 92.48%, of which 75.1% to 81.31% were uniquely mapped. Details of transcriptome sequencing and alignment with the reference genome were shown in Table 1.

**Table 1:** Summary of the sequence data from RNA sequencing.

Samples	Replication	Clean reads	Clean bases	GC Content (%)	Q30(%)	Mapping Ratio (%)	Uniq Mapped Reads	Uniq Mapping Ration (%)
DS(0 day)	1	21, 340, 529	6, 957, 770, 010	45. 04	94. 84	91. 72	18, 785, 808	80. 74
	2	23, 668, 091	7, 672, 474, 678	45. 85	94. 73	92. 32	20, 158, 384	78. 63
	3	28, 352, 632	9, 145, 783, 980	45. 39	93. 86	92. 48	24, 928, 120	81. 31
DS(7-day)	1	17, 561, 530	6, 071, 692, 562	45. 31	94. 24	86. 65	15, 455, 767	76. 26
	2	20, 034, 502	6, 715, 926, 858	45. 74	93. 98	89. 35	17, 794, 494	79. 36
	3	20, 645, 249	6, 854, 475, 054	45. 44	94. 01	90. 25	18, 035, 140	78. 84
DS(14-day)	1	19, 042, 765	6, 508, 686, 710	45. 04	94. 88	87. 45	16, 810, 766	77. 20
	2	20, 079, 677	6, 660, 559, 720	44. 82	94. 34	90. 24	17, 658, 723	79. 36
	3	21, 891, 997	7, 261, 979, 340	45. 03	94. 78	90. 21	19, 380, 278	79. 86
DS(21-day)	1	19, 013, 668	6, 625, 166, 840	45. 18	94. 14	85. 71	16, 659, 975	75. 10
	2	38, 338, 429	12, 958, 568, 496	45. 25	94. 81	88. 36	33, 886, 729	78. 10
	3	21, 211, 202	7, 009, 506, 402	45. 03	93. 59	90. 54	18, 659, 954	79. 65
DT(0-day)	1	40, 161, 191	13, 040, 250, 156	45. 68	94. 4	92. 28	34, 995, 247	80. 41
	2	31, 602, 635	10, 341, 203, 072	45. 5	94. 54	91. 39	27, 781, 549	80. 34
	3	58, 902, 201	19, 240, 825, 954	45. 62	94. 97	91. 44	52, 028, 989	80. 77
DT(7-day)	1	20, 268, 740	6, 727, 469, 924	44. 86	94. 15	90. 25	17, 800, 558	79. 26
	2	20, 835, 103	6, 805, 129, 560	45. 19	94. 28	91. 67	18, 266, 797	80. 37
	3	20, 350, 607	6, 649, 290, 422	45. 02	94. 03	91. 70	17, 907, 202	80. 69
DT(14-day)	1	20, 659, 825	6, 828, 061, 186	44. 89	93. 9	90. 58	18, 128, 096	79. 48
	2	20, 686, 964	6, 974, 982, 690	44. 86	94. 01	88. 71	18, 072, 818	77. 50
	3	21, 284, 737	6, 983, 048, 438	45. 11	93. 57	91. 21	18, 577, 765	79. 61
DT(21-day)	1	20, 609, 235	6, 718, 960, 908	45. 36	94. 39	91. 79	18, 065, 356	80. 46
	2	17, 567, 823	5, 801, 000, 172	45. 49	94. 39	90. 63	15, 542, 183	80. 18
	3	20, 713, 256	6, 775, 792, 422	45. 39	94. 51	91. 46	18, 160, 901	80. 19

**Fig 2:**
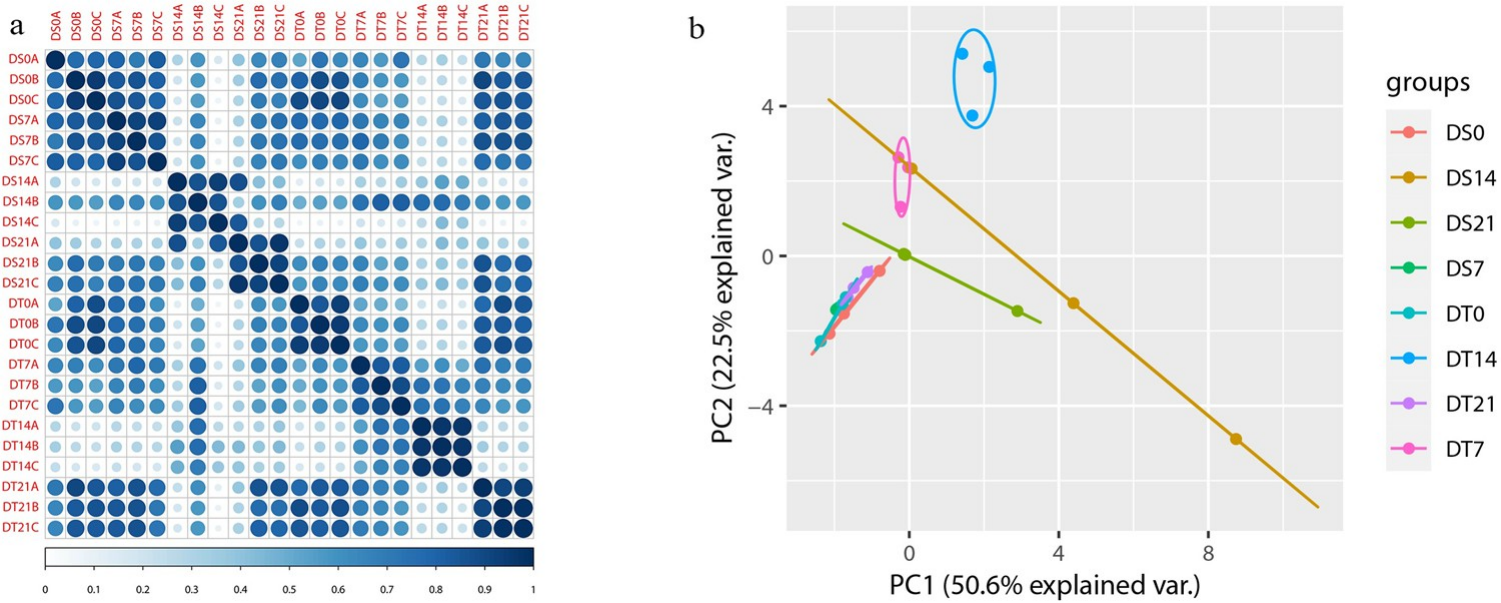
Transcriptional relationship among twenty-four samples. (**a)** Person correlation coefficient analysis of transcriptome data from two sunflower varieties under drought stress. (**b)**Principal component analysis of the data showing the variation due to genotype and treatment.

To assess the correlations of the 24 samples, we then conducted a clustering analysis. The transcriptome data of three biological replicates from each drought stress time-point exhibited a good correlation (Fig 2A). Then, the PCA was applied for dimensionality reduction using the first principal component (PC1) and second principal component (PC2) to analyze the similarity between each replicate(Fig 2B). The results showed tight aggregation between the three replicates of each sample except DS14.

### Differentially expressed gene analysis

A total of 24,234 DEGs were obtained at three drought stress time points for two varieties, including 11,483 in DS and 18,922 in DT (S1 Fig, S3 Table).

**Fig 3:**
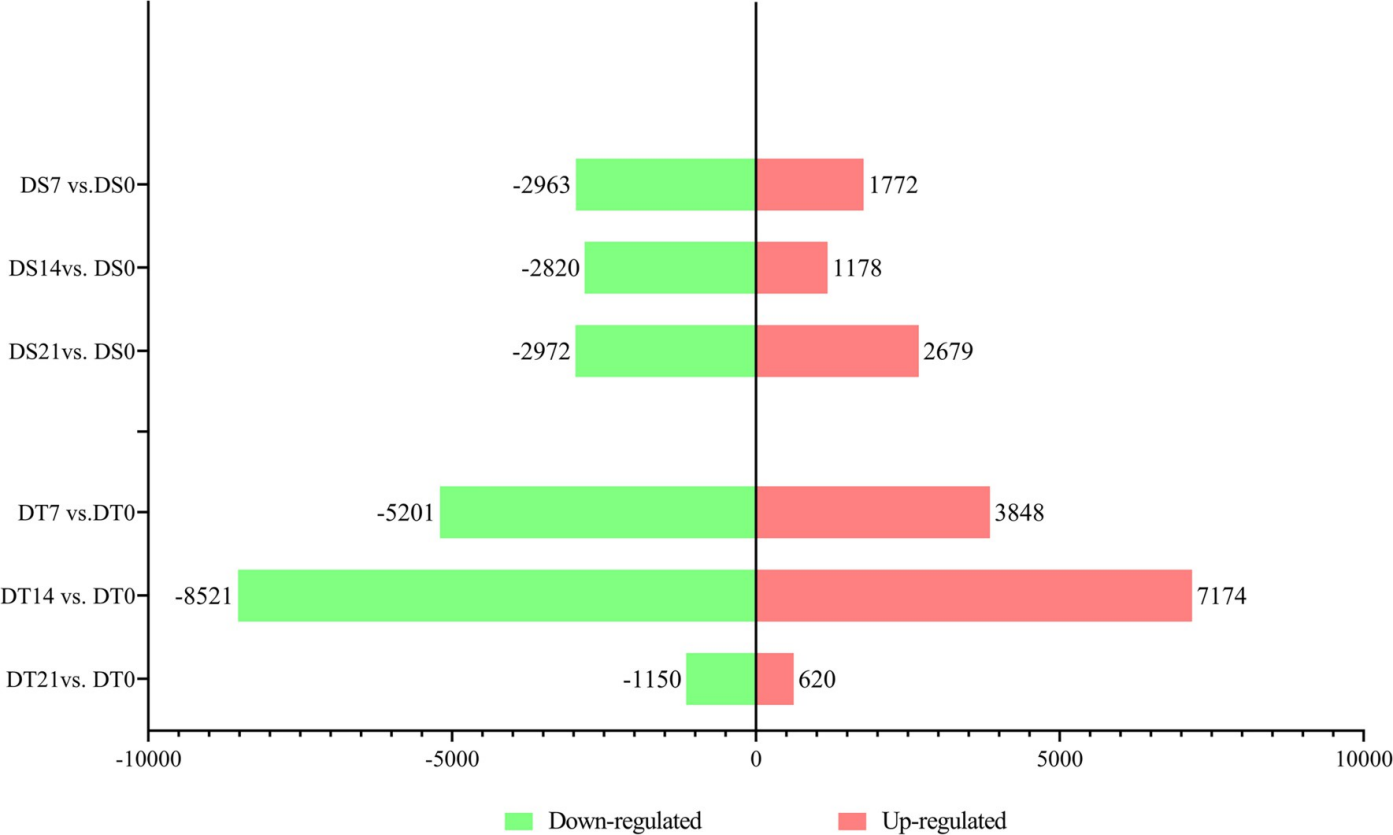
Histogram of DEGs in DS and DT under three drought stress time.

The number of down-regulated genes was higher than up-regulated genes at the same drought stress time (Fig 3). Up-regulation DEGs in DS was 1,772 at 7 day which reduced to 1,178 at 14 day and increased to 2,679 at 21 day. Meanwhile, the number of down-regulated DEGs in DS was decreased from 2,963 at 7 day to 2,820 at 14 day, then increased to 2,972 at 21 day. In contrast, the number of DEGs in DT was dramatically changed with the duration of the drought stress. The up-regulated DEGs were increased from 3848 to 7,174 at 7 day to 14 day, and decreased to 620 at 21 day, while the down-regulated DEGs were increasing from 5,201 to 8,521 at 7 day to 14 day, then decreasing to 1,150 at 21 day.

### Function annotation and classification

To reveal the functional differences of DEGs, GO enrichment analysis was carried out using the 24,234 DEGs. The GO terms were subdivided into three categories: biological process (BP), cellular component (CC), and molecular functions (MF) (S2 Fig) (S4 Table).

To make the results more concise, we compared the number of genes enriched in the common GO-term across varieties, and only the most significant BP GO-terms were analyzed.

**Fig 4:**
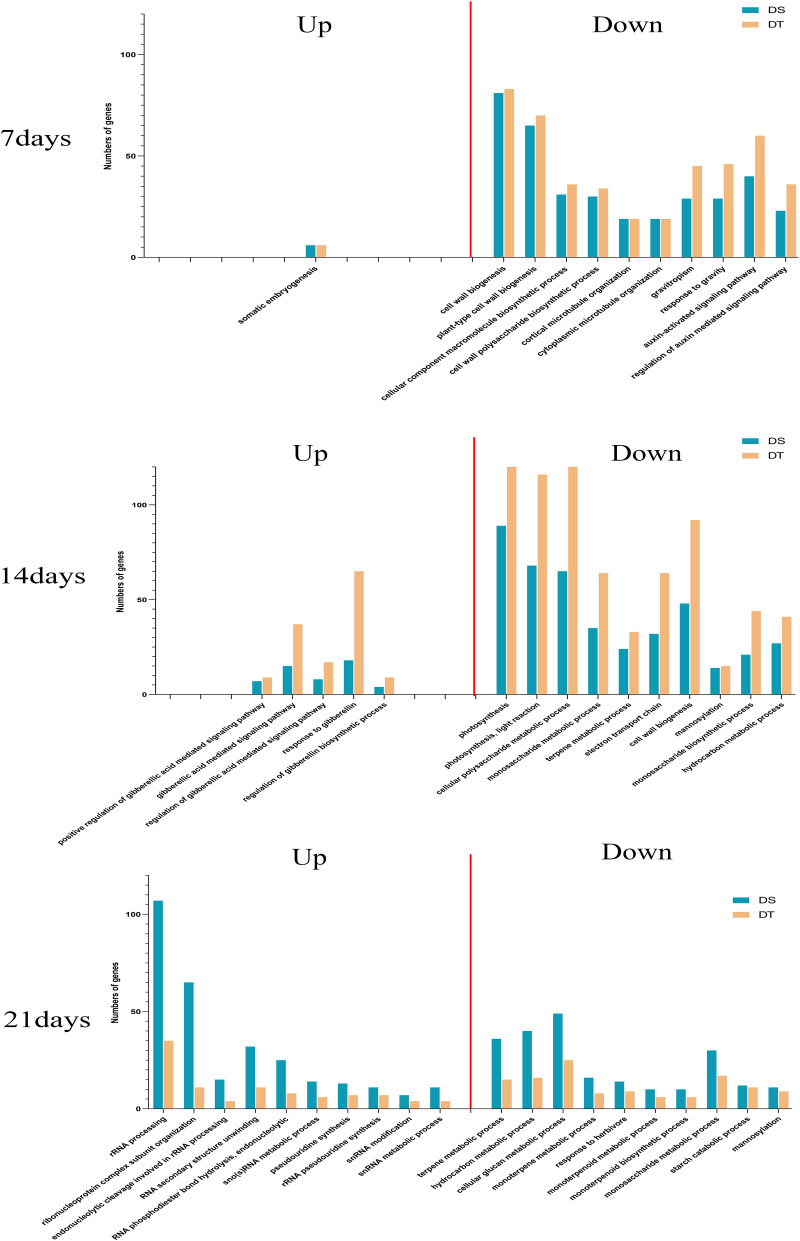
GO analysis for significant DEGs. Graphical representation of number of DEGs enriched in common biological process GO-terms in DS comparative to DT at all three-time intervals.

For up-regulated DEGs, somatic embryogenesis (GO:0010262) was the only common GO-terms at 7 day. At 14 day, five common GO-terms (GO:0009939, GO:0009740, GO:0009937, GO:0009739, GO:0010371) were all related to gibberellin, and the number of genes enriched to the same GO-terms in DT was higher than that in DS. However, the opposite was observed at day 21, when the number of genes enriched to the common GO terms was higher for DS than for DT, and a total of 22 common GO-terms were enriched at 21 day under drought stress, most of them were related to rRNA and snRNA, such as rRNA processing (GO:0006364), endonucleolytic cleavage involved in rRNA processing (GO:0000478), and snRNA modification (GO:0040031). For down-regulated DEGs, there were 15,33, and 18 common GO-terms enriched at day 7, 14, and 21, respectively. DEGs at 7 day were mainly enriched in cell wall (GO:0042546, GO:0009832, GO:0070592), microtubule organization (GO:0043622, GO:0031122), and auxin signal-related (GO:0009734, GO:0010928) GO-terms. On day 14, the down-regulated genes were mainly enriched in photosynthesis (GO:0015979, GO:0019684), sugar synthesis/metabolism (GO:0044264, GO:0005996, GO:0046364) related GO-terms. Among the top 10 go-terms enriched at day 21, 4 of them were also detected at day 14 (GO:0042214, GO:0120252, GO:0005996, GO:0097502), and the GO-terms enriched in this period were mainly processes related to sugar metabolism (GO:0006073, GO:0006073, GO:0005996, GO:0005983, GO:0097502) and terpene metabolism (GO:0042214, GO:0043692, GO:0016098, GO:0016099) (Fig 4, S5 Table), indicates the important role of these biological processes in the middle and late stages of sunflower under drought stress.

### KEGG pathways enrichment analysis of the DEGs

**Fig 5:**
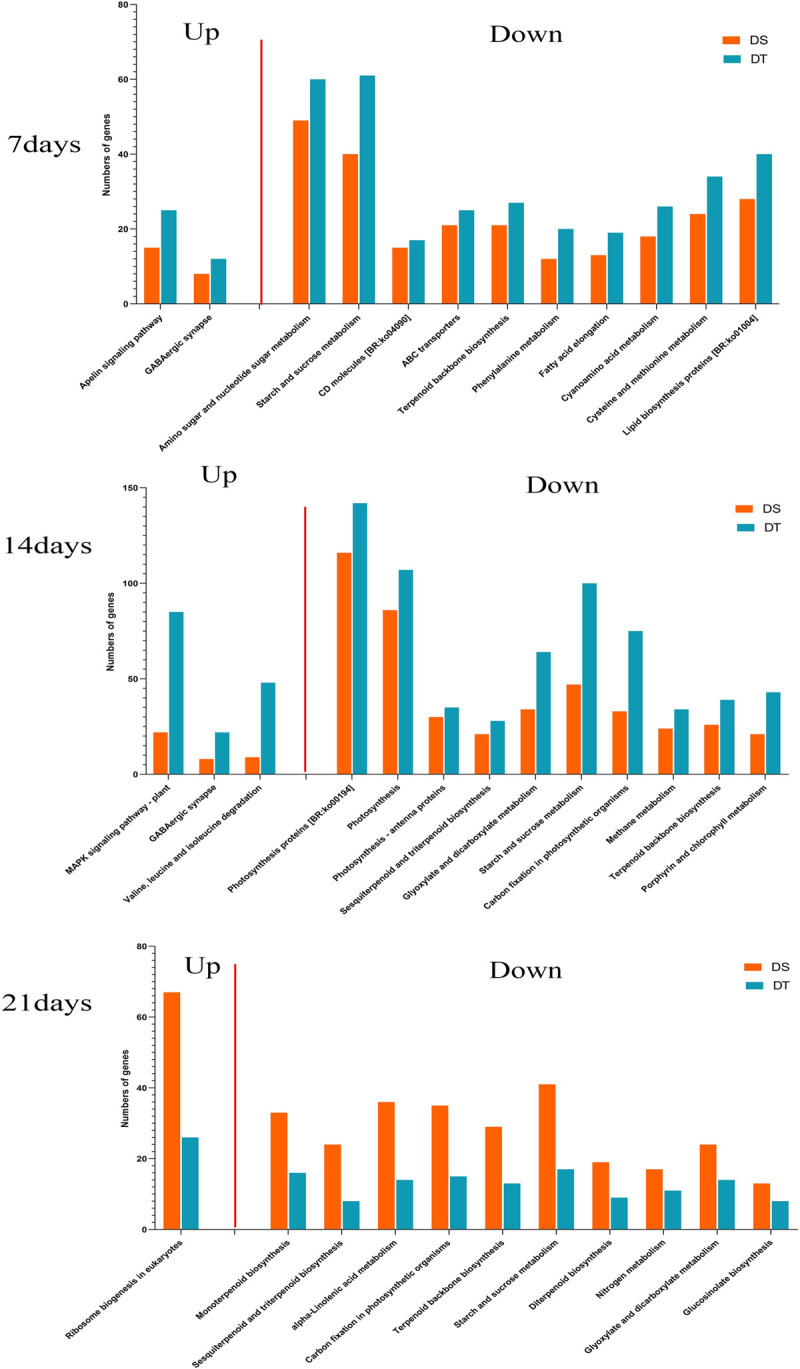
Pathway analysis for significant DEGs. Graphical representation of number of DEGs enriched in common KEGG pathways in DS comparative to DT at all three-time intervals.

KEGG enrichment analysis was conducted to identify pathways for these DEGs. (S3 Fig, S6 Table). We compared the differences in the number of genes enriched in the common KEGG pathways between DS and DT at three times (Fig 5, S7 Table).

Overall, the number of up-regulated genes was less than that of down-regulated genes, so fewer pathways were enriched. In addition, DT enriched more genes in the same metabolic pathway than DS on days 7 and 14, while the reverse was found on days 21.

For up-regulated DEGs, GABAergic synapse pathway was enriched at both day 7 and day 14. In addition to this, on day 14, genes were also enriched to the MAPK signaling pathway, Valine, leucine, and isoleucine degradation, while Ribosome biogenesis in eukaryotes was the only common pathway enriched by up-regulated DEGs at day 21.

For down-regulated DEGs, there were 12, 26, and 15 common pathways were enriched on days 7, 14, and 21, respectively, some of which were already reported to be drought-related pathways. Major pathways on day 7 were Amino sugar and nucleotide sugar metabolism, Starch and sucrose metabolism, ABC transporters, and Terpenoid backbone biosynthesis, while on day 14, more photosynthesis-related pathways were obtained, such as Photosynthesis proteins, Photosynthesis, and Photosynthesis- antenna proteins. The top pathways on day 21 were Monoterpenoid biosynthesis, Sesquiterpenoid and triterpenoid biosynthesis and alpha-Linolenic acid metabolism.

Notably, among the top 10 pathways, 2 pathways (Starch and sucrose metabolism, Terpenoid backbone biosynthesis), were enriched at 3 time points simultaneously, and 3 pathways (Sesquiterpenoid and triterpenoid biosynthesis, Glyoxylate and dicarboxylate metabolism, and Carbon fixation in photosynthetic organisms) were enriched at both 14 and 21 day. These pathways may be closely related to the drought resistance of sunflowers.

### TFs genes in response to drought stress

TFs play a key role in plant response to abiotic stress. A total of 1,311 presumed TFs were identified in the present study from all 24,234 DEGs (S8 Table), among which bHLH (110 genes), AP2/ERF-ERF (89 genes), MYB (89 genes), C2H2(83 genes), NAC (83 genes), WRKY (79 genes), HB-HD-ZIP (57 genes), MYB-related (57 genes), bZIP (56 genes), and HSF (24 genes) were the top 10 transcription factor families with the high abundance.

**Fig 6:**
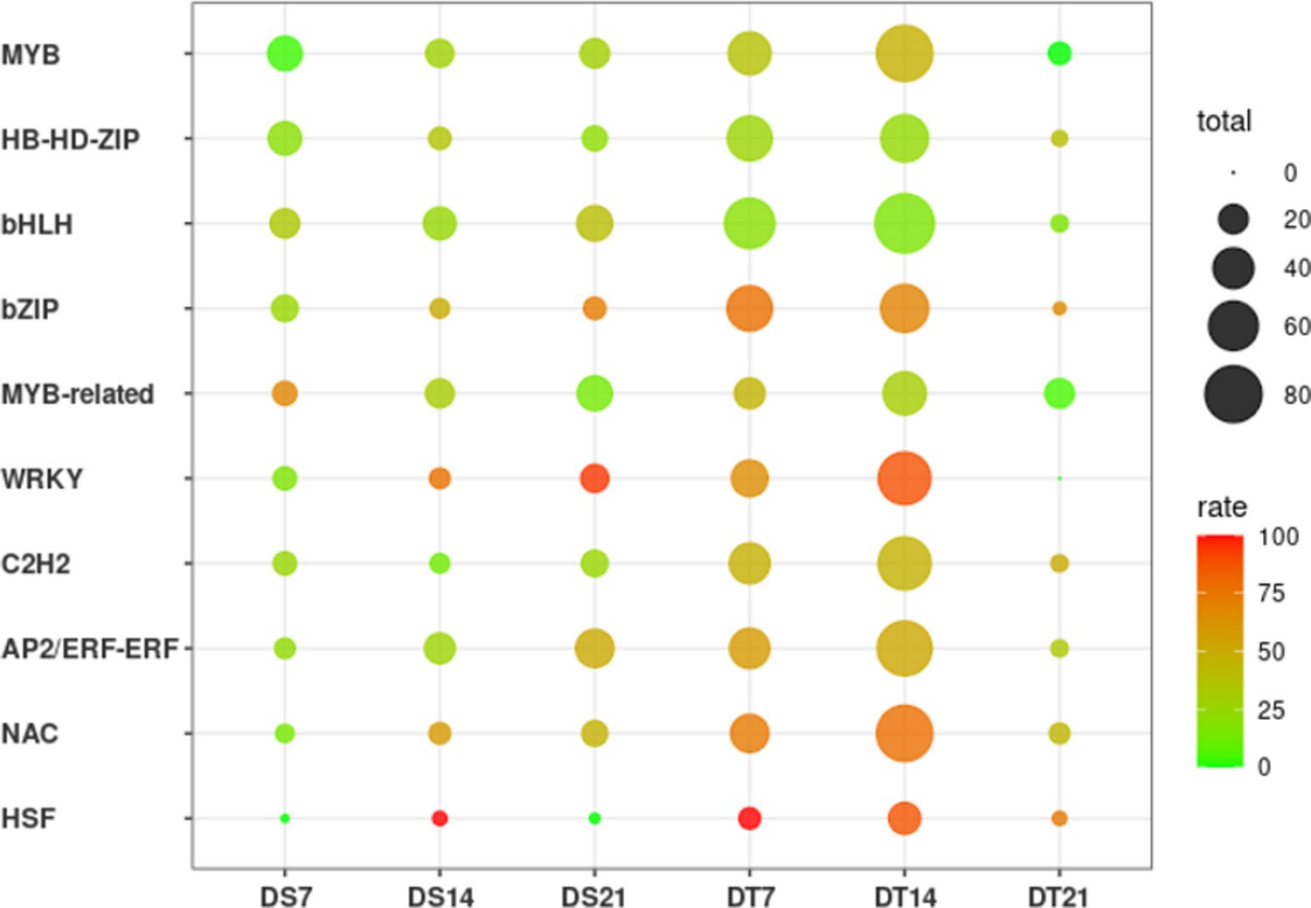
Ten transcription factor families with the largest number of genes. The size of the circle represents the total number of transcription factors, and color represents the proportion of up/down-regulated genes in total genes, The redder the color, the more genes are up-regulated, and the greener the more genes are down-regulated.

The total number and the proportion of up/down-regulated genes in each TF family among different varieties and drought stress times were demonstrated in Fig 6.

A large number of TFs genes were expressed on days 7 and 14 in DT, especially on day 14, when the proportion of TFs ranged from 30.4% (HB-HD-ZIP) to 53.3% (HSF) of the total number. In addition, genes belonging to MYB, HB-HD-ZIP, and bHLH families had higher down-regulation rates in both varieties. The down-regulation rate of the MYB transcription factor was 96.43% and 100% in DS7 and DT21, respectively. The down-regulation rate of HB-HD-ZIP ranged from 60.00% in DT21 to 81.77% in DS7, while the down-regulation rate of the bHLH transcription factor ranged from 60.00% in DS21 to 83.91% in DT14. Meanwhile, genes belonging to the bZIP, WRKY, AP/ERF, NAC, and HSF TFs families had a relatively high up-regulation rates, especially in DT14, when both the total number and the up-regulation rate reached the highest level. A total of 56, 68, 74, 78, and 24 genes belonging to bZIP, WRKY, AP/ERF, NAC, and HSF families were identified in DT14, with up-regulation rates of 69.64%, 88.24%, 52.70%, 78.21%, and 87.50%, respectively.

### Mapman metabolic process analysis

MapMan was used to provide an overview of the DEGs at the metabolic level. The "metabolic overview" panel provides metabolic overview, which is divided into CHO metabolism, lipids metabolism, secondary metabolism, amino acids, while the “drought stress” panel showed in detail the expression profile of DEGs related to hormone signaling, TFs, and redox state under drought stress. In general, the results of Mapman analysis, such as metabolism profile of “cell wall”, “terpen”, “starch and sucrose” and transcript factors were in good consistency with the results of GO, KEGG, and TFs analysis, which proved the reliability of our results.

**Fig 7:**
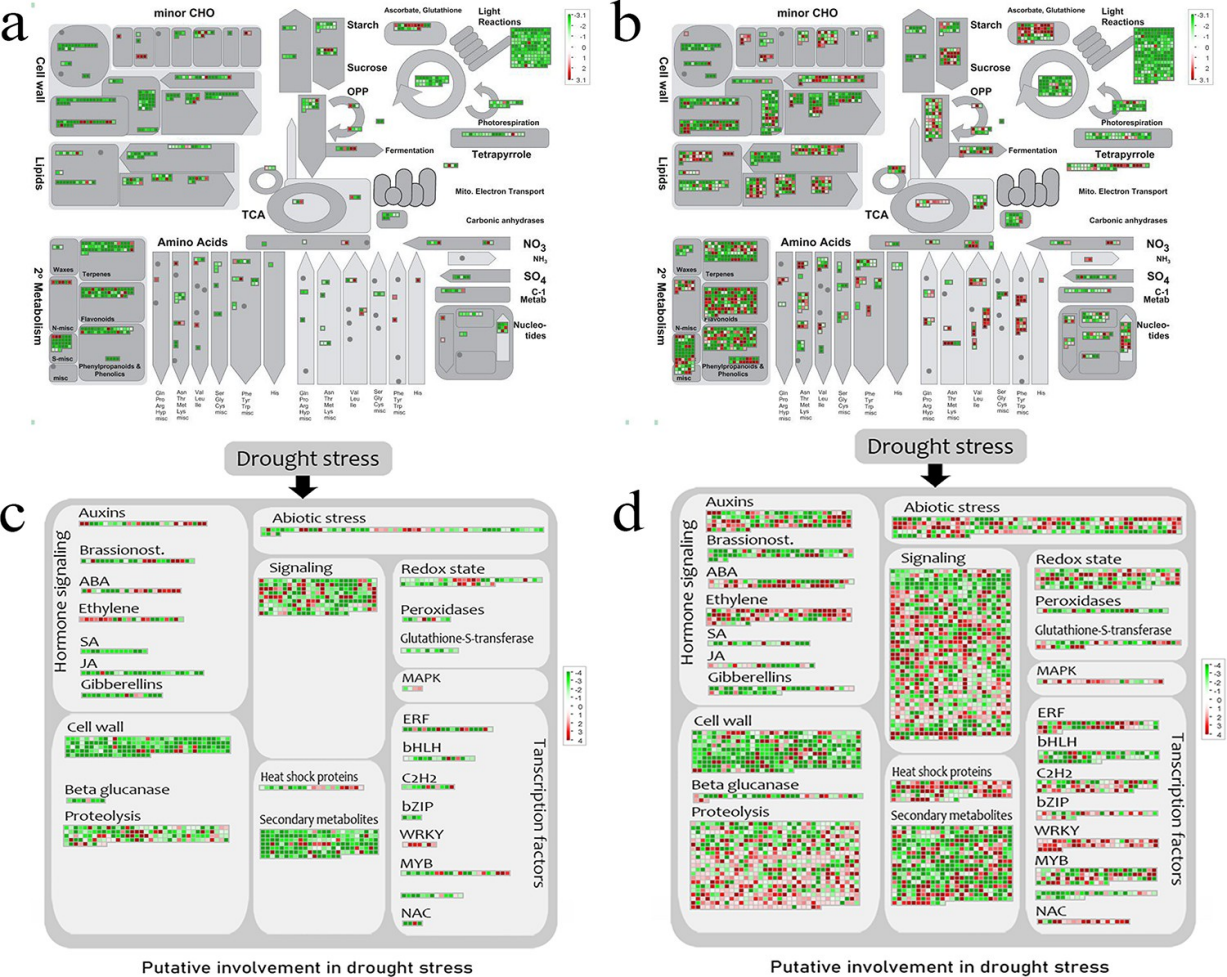
MapMan visualization of drought stress-responsive DEGs in DS and DT under drought stress. Each square represents a gene, red means up-regulated, green means down-regulated. (a)Metabolism overviews of DS at 14-days of drought stress. (b) Metabolism overviews of DT at 14-days of drought stress. (c) Abiotic stress metabolism pathway of DS after 14-days of drought-stress. (d) Abiotic stress metabolism pathway of DT after 14-days of drought-stress.

In addition, Mapman revealed that the most significant differences in gene number and expression level were observed at 14-days of drought stress, so our analysis focused on the results at day 14 (Figs 7, S4 and S5). Due to the important effect of plant hormones on abiotic stress, we investigated the different expressions of seven plant hormones, including Auxin, Brassinosteroid (Br). abscisic acid (ABA), ethylene, salicylic acid (SA), jasmonates (JA), and gibberellins.

There were 129, 64, 58, 99, 21, 22, and 45 genes identified in DT that belonged to Auxin, Br, ABA, ethylene, SA, JA and gibberellins, while 27, 24, 21, 22, 14, 26, and 17 genes belonged to the above seven phytohormones in DS, respectively. It can be seen that the number of DS is lower than that of DT, except for JA. In addition, we found that the proportion of up/down-regulated genes in JA and SA were significantly different between the two varieties, with 19% of the SA genes up-regulated in DT and all of these genes down-regulated in DS; the proportion of up-regulated JA genes in DT was 45.45%, while in DS it was only 3.85%.

### WGCNA identifies candidate modules associated with seedling drought-resistance traits

**Fig 8:**
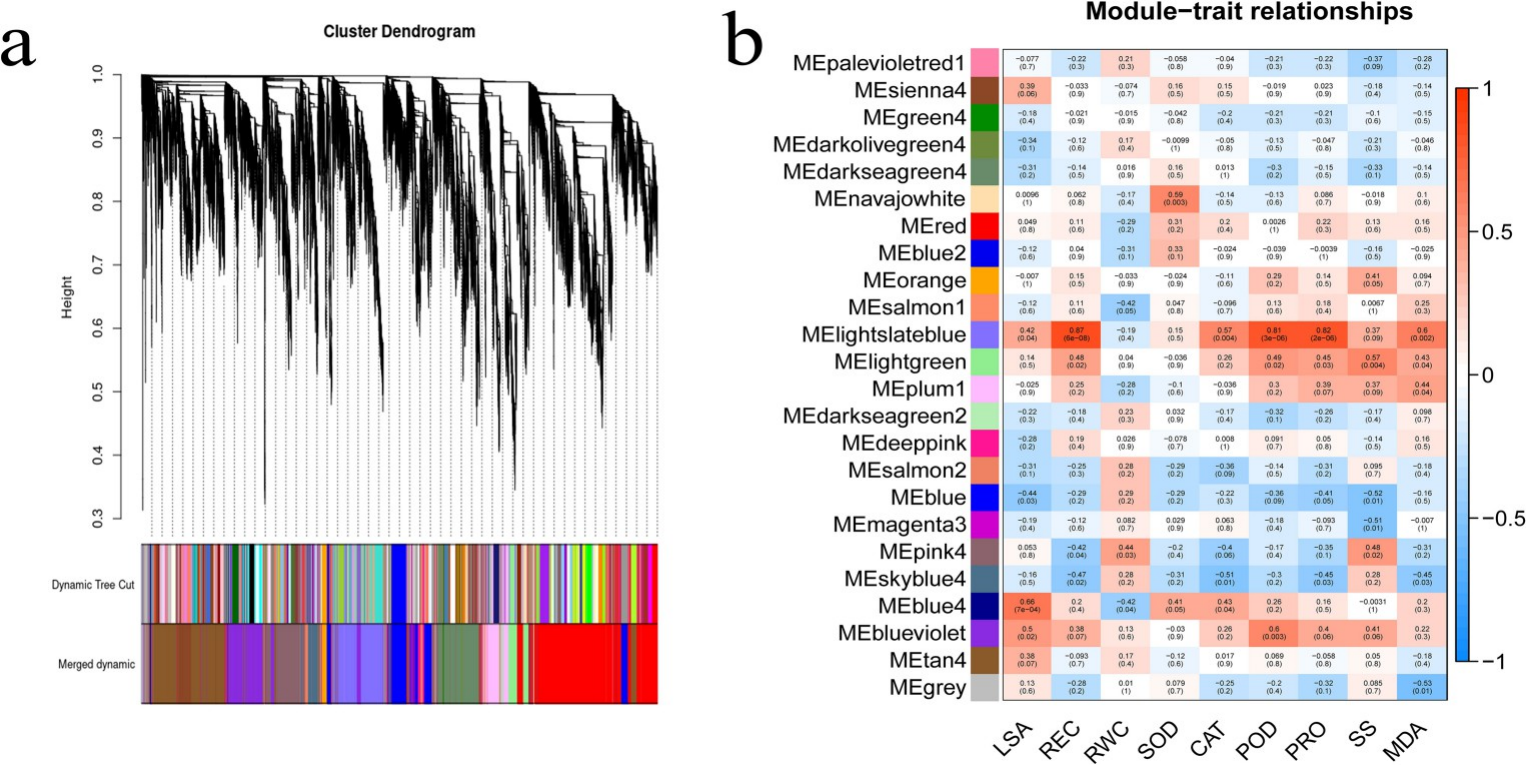
WGCNA co-expression network and module-trait correlation analysis. (a) Hierarchical cluster tree showing co-expression modules identified by the Dynamic Tree Cut method. Each leaf, that is a short vertical line, corresponds to a gene. Branches of the dendrogram group together densely interconnected constitute modules and are labeled with different colors. Genes with highly co-expression level (correlation≥ 0. 75) were merged to one module, 24 modules were obtained in total. (b) Correlations of physiological traits with WGCNA modules. Each row corresponds to a module The columns correspond to nine physiological traits. The color of each cell indicates the correlation coefficient between the module and traits, Red represents positive correlation and blue represents negative correlation. (The top number in the cell represents the correlation coefficient, and bottom one in parentheses represents the P value).

All 24,234 DEGs were retained for WGCNA unsigned co-expression network analysis. After clustering the samples, an outlier (DS14C) was removed in subsequent analysis (S6 Fig). The soft threshold power of 14 (β = 14) was selected according to the preconditions of approximate scale-free topology (S7 Fig). The analysis identified twenty-four distinct co-expression modules(labeled with diverse colors)shown in the dendrogram, of which blue4 module(containing 73 genes)was positively associated with LSA, with correlation coefficient(r) of 0.66(p = 7×10^−4^), while lightslateblue modules (containing 2,178 genes) positively associated with the REC, CAT, POD, Pro, and MDA (Fig 8), with correlation coefficient(r) of 0.87 (p = 6 × 10^−8^), 0.57(p = 4 × 10^−3^), 0.81(p = 3× 10^−6^), 0.82(p = 2 × 10^−6^) and 0.6(p = 2 × 10^−3^), respectively. This phenomenon may indicate possible correlations between genes that determine different drought-resistant traits.

### GO and KEGG enrichment analysis of genes in each modules

**Fig 9:**
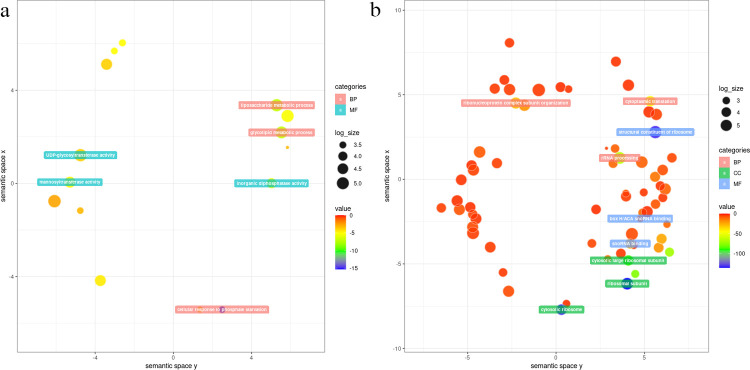
GO analysis results of each module. (a)Genes in blue 4 module. (b)Genes in lightlateblue module.

Genes in the blue 4 module were enriched in 20 GO-terms, after de-redundancy, 15 GO-terms were obtained, of which 11 in BP and 4 in MF. The top three significant GO-terms ranked by p-value for the BP were GO:0016036(cellular response to phosphate starvation), GO:0006664(glycolipid metabolic process), and GO:1903509(liposaccharide metabolic process). Genes in the lightslateblue module were enriched in 65 de-redundancy GO-term, including 30 in BP, 17 in CC, and 18 in MF. The top three significant GO-terms for the BP were GO:0006364 (rRNA processing), GO:0002181 (cytoplasmic translation), and GO:0071826 (ribonucleoprotein complex subunit organization) (Fig 9, S9 Table).

**Fig 10:**
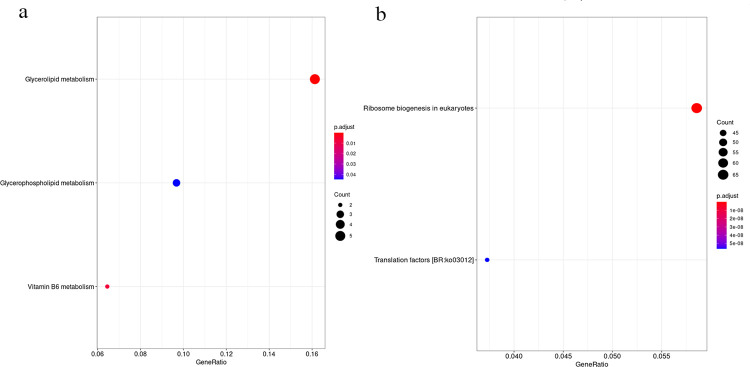
KEGG pathway analysis results of each module. (a) Genes in blue 4 module. (b) Genes in lightlateblue module.

Pathway analysis revealed that genes in the blue4 module were enriched in glycerolipid metabolism, vitamin B6 metabolism, and glycerophospholipid metabolism, while in lightslateblue module, genes were enriched in ribosome biogenesis in eukaryotes and translation factors (Fig 10, S10 Table).

### Network visualizing

**Fig 11:**
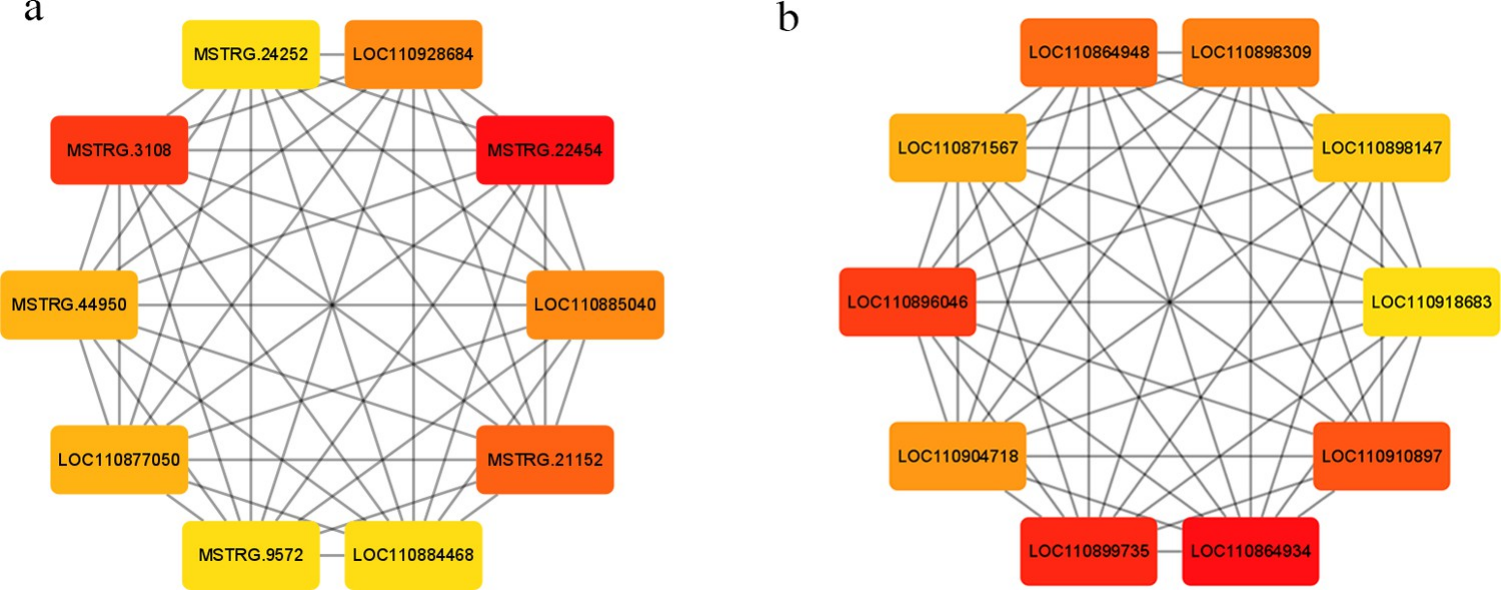
The top 10 genes in each module are calculated by MCC algorithm of cytohubba. (a)Top 10 genes in blue 4 module. (b)Top 10 genes in lightlateblue module.

The top 10 hub genes in each module were identified via the cytoHubba plug-in (Fig 11, Table 2). Genes in the blue 4 module have different functions, such as caffeoyl-CoA O-methyltransferase (LOC110877050), monogalactosyldiacylglycerol (MGDG)synthase (MSTRG. 21152, LOC110928684), and two protein kinase(PK) genes were identified LOC110885040(Epidermal growth factor-like domain, belongs to RLK-Pelle_WAK family) and MSTRG. 22454(Receptor-like cytosolic serine threonine-protein kinase RBK1, belongs to RLK-Pelle_RLCK-VI family). while gene annotations in lightslateblue are all about ribosomes such as Belongs to the universal ribosomal protein uL13 family (LOC110864934), ubiquitin-40S ribosomal protein (LOC110896046), and ribosomal protein (LOC110904718).

**Table 2:** Annotaions of top 10 genes ranked by MCC algorithm.

Module	Gene ID	eggNOG free text desc.	iTAK	Familes
blue4	MSTRG. 22454	Receptor-like cytosolic serine threonine-protein ki-se RBK1	PK	RLK-Pelle_RLCK-VI
	MSTRG. 21152	Monogalactosyldiacylglycerol (MGDG) synthase	-	-
	LOC110928684	Monogalactosyldiacylglycerol (MGDG) synthase	-	-
	LOC110885040	Epidermal growth factor-like domain.	PK	RLK-Pelle_WAK
	LOC110877050	caffeoyl-CoA O-methyltransferase	-	-
	MSTRG. 44950	Pentatricopeptide repeat-containing protein	-	-
	MSTRG. 9572	phosphatidylinositol dephosphorylation	-	-
	LOC110884468	Glycosyl transferase 4-like	-	-
	MSTRG. 24252	-	-	-
lightslateblue	LOC110864934	Belongs to the universal ribosomal protein uL13 family	-	-
	LOC110899735	Belongs to the eukaryotic ribosomal protein eS1 family	-	-
	LOC110896046	ubiquitin-40S ribosomal protein	-	-
	LOC110910897	Belongs to the eukaryotic ribosomal protein eS7 family	-	-
	LOC110864948	60s ribosomal protein	-	-
	LOC110898309	Belongs to the universal ribosomal protein uS12 family	-	-
	LOC110904718	ribosomal protein	-	-
	LOC110871567	Ubiquitin-binding WIYLD domain	-	-
	LOC110898147	Belongs to the universal ribosomal protein uS7 family	-	-
	LOC110918683	60s ribosomal protein	-	-

### Verification of RNA-seq data by RT-qPCR

**Fig 12:**
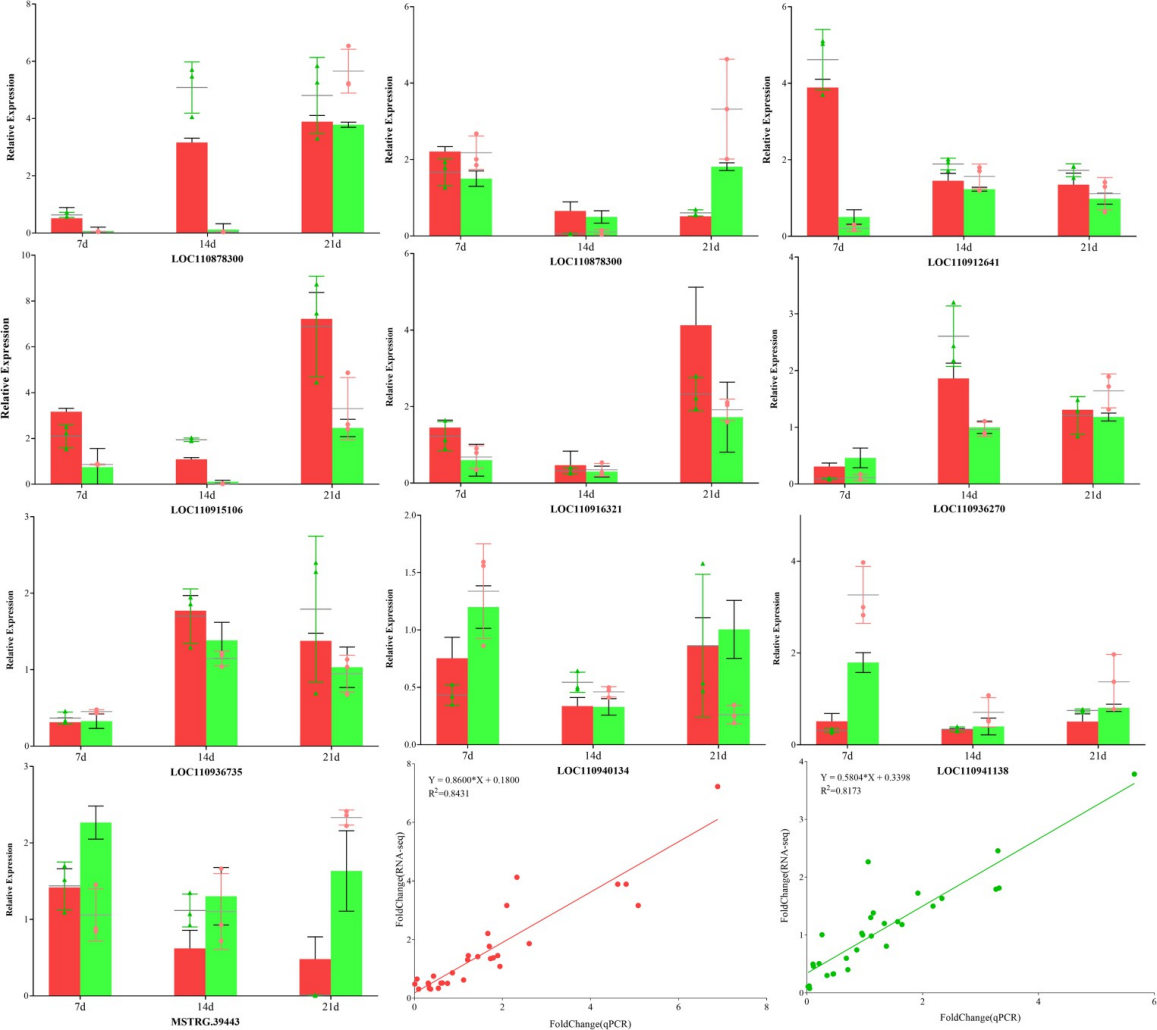
RT-qPCR analysis of 10 drought-stress related DEGs during various stages of drough stress. Bars with standard errors represent relative expression level determined by the ratio of sample average FPKM to control. The scatter plot on the bar graph represents the corresponding relative expression level determined from three independent biological replicates by RT-qPCR using the 2^-△△Ct^ method. Correlation between RT-qPCR and RNA-seq for select DEGs is also shown in the end.

To validate the accuracy of RNA-seq data, RT-qPCR was performed for 10 genes randomly selected from DEGs. Linear regression analysis showed that the correlation between RNA-seq and RT-qPCR data was significantly positive with a correlation coefficient(*r*^2^) of 0. 8431 and 0. 8173 in DS and DT, respectively, endorsing our RNA-seq data were reliable (Fig 12).

## Discussion

Sunflower is an economically important oilseed crop and shared 9% of the world oilseed market [48]. It is considered a medium drought-resistant crop, and with the extension of cultivation to arid areas, drought has become one of the most important factors affecting its yield [49,50]. Therefore, revealing the molecular mechanism of drought tolerance in sunflowers is important for drought-resistant genetic breeding.

Previous studies have shown that the effects of drought on sunflowers are related to the duration of stress and genotype, so we performed transcriptome sequencing and bioinformatics analysis using two inbred lines with different drought resistance under different stress times.

### Differential performance of physiological and biochemical indexes

Early drought stress reduces sunflower leaf area, which reduces the radiation use efficiency and photosynthetic activities [51,52]. Our results illustrated that early drought stress led to a decrease in LSA, but DT could recover to normal levels more quickly, especially at 21 day, when LSA of DT was significantly higher than that of DS.

Cell membranes are one of the primary targets of drought stress, and drought stress leads to cytomembrane disruption and electrolyte leakage, resulting in increased REC [53]. In this study, the REC continued to increase with the extension of drought stress time, while DS was slightly higher than DT, indicating that the cell membrane of DT maintained relatively better integrity.

Reactive oxygen varieties(ROS), including singlet oxygen (O_2_^-^), hydrogen peroxide (H_2_O_2)_, and hydroxyl radicals(**·**OH), is a important class of signaling molecules that maintain relative balance in the plant under normal conditions [5]. Under drought stress, the decrease of photosynthesis leads to the decrease of electron acceptor NADP^+^ in the electron transport chain, and a large number of electrons leak to O_2_, generating excessive ROS [54,55], which can seriously damage plants by increasing lipid peroxidation, protein degradation, and even cell death [56]. In addition, excessive production of ROS varieties increases the level of MDA, which is considered to be an indicator of oxidative damage. To minimize the effects of oxidative stress, plants have evolved complex ROS scavenging enzymes system, such as SOD, POD, and CAT [57], which cooperate with each other to reduce the oxidative damage of cells. SOD converts O_2_^-^ to H_2_O_2_ through dismutation reaction, and then H_2_O_2_ is finally eliminated by POD and CAT. Our results showed that two antioxidant enzymes (CAT, POD) and MDA increased with the continuation of drought stress, and the enzyme activities were higher in DS than DT at different time points. indicating that DS suffered more oxidative damage, while SOD decreased at day 21, which may indicate a decrease in O_2_^-^ production at day 21.

Plant leaves lose water through transpiration, and the failure to replenish water under drought stress leads to a decrease in cell water content, cell membrane contraction, and reduced cell membrane integrity [58]. Plants maintain their water balance by producing different osmolytes/solutes, i.e. osmoregulation. These solutes, such as Pro and SS, protect cell structure and function by maintaining cell turgor and other physiological mechanisms under water deficit conditions [59,60]. An increase in the concentration of Pro and SS content and decreased RWC content in the leaves of sunflowers under drought stress have been reported [61,62].

In the present study, as drought stress sustained, RWC continued to decrease, and DT was higher than DS at different time points, indicating that DT maintained a higher cell turgor. The Pro content continued to increase, and it is slightly higher in DS than in DT at the same time. While in the early stage of drought stress, SS of both varieties decreased significantly, then gradually increased, and DS was higher than DT at day 21, indicating that DS was still affected by intense stress at day 21. Al Hakimi A. et al. suggested that SS content can more accurately identify drought tolerance in durum wheat (*Triticum durum Desf*.) compared to Pro [63]. However, we found a higher correlation between Pro content and stress time in sunflower, which may better reflect the osmotic adjustment intensity than SS.

In general, our results showed that all the 9 selected indicators were affected under drought stress with different degrees, and DT was more tolerant to drought stress than DS.

### Drought stress results in more genes being down-regulated

The number of DEGs under drought reflects the response intensity of the crop. Escalante et al. observed that there were more DEGs in drought-sensitive sunflower lines compared to drought tolerance lines under drought stress [19]. In addition, the number of up-regulated genes was higher than down-regulated genes in both Liang`s [23] and Escalante’s [19] studies in sunflower. In the present study, the number of DEGs was higher in DT than in DS, except on day 21, while the number of up-regulated DEGs was lower than the number of down-regulated DEGs. Although the analysis results of DEGs numbers are not quite consistent with previous studies in sunflower, they are highly consistent with a study in maize [64]. The reasons for these differences may be related to stress intensity, stress duration, and genotype. In particular, the substantially decreased DEGs in DT at day 21 day deserve further investigation.

### Important biological processes in drought stress response in sunflower

GO analysis results showed that some important biological processes exhibit temporal specificity, and several important GO-terms such as cell wall, gibberellic acid, Photosynthesis, rRNA processing, and Terpene metabolism process were only found at specific time points of drought stress.

Plants resist osmotic stress under drought by increasing cell wall elasticity and thus maintaining cell turgor [65]. However, in previous reports on sunflower, the increase in cell wall elasticity was lost and even decreased during subsequent water deficit [66]. Later, a transcriptome analysis showed that down-regulated genes were enriched to cell wall synthesis-related pathways, explaining the previous phenomenon from the genetic level [19]. Consistent with previous findings, our results also demonstrate the down-regulation of genes related to the biological process of cell wall synthesis under drought stress, suggesting that increasing the elasticity of the cell wall may not be the main approach of sunflower to cope with drought.

Gibberellic acid was functioned as plant growth regulator, increases the amount of sucrose phosphate synthase and fructose-1, 6-biphosphatase, and inhibit oxidative stresses in plant. Exogenous addition of gibberellin enhanced resistance tolerance in sunflower [67]. Our study found that gibberellin-related genes were up-regulated at mid-drought stress (14 day), indicating an important role of endogenous gibberellin in sunflower response to drought stress. Although the role of endogenous gibberellin in sunflower drought resistance has not been reported at present, our findings were consistent with the research of maize [68].

Drought could affect photosynthesis by the diffusion limitations through the stomata and the mesophyll and/or by alterations in photosynthetic metabolism [69]. A previous study detected an increase in the expression level of photosynthesis-related genes [22] under drought stress, while another study demonstrated that most of the down-regulated genes were involved in photosynthesis [19]. Our findings are consistent with the latter, with down-regulation of gene enrichment to photosynthesis-related biological processes at day 14.

GO-terms associated with rRNA processing were found in other previous research of drought stress [70]. The up-regulated GO category contained “RNA metabolic processes” was identified by the research of Sebastián Moschen et al in sunflower [22]. In the present study, we also found that up-regulated genes were enriched to rRNA-related GO-terms under 21 day of drought stress, such as “rRNA processing”, “ribonucleoprotein complex subunit organization”, and “RNA secondary structure unwinding”, indicating that the effect of late drought stress on cellular gene levels.

Terpene accumulation is related to the function of resistance to environmental stresses [71]. However, the down-regulation of genes enriched in “terpene metabolism process” in our study is contrary to the previous one [22]. Due to the role of terpenes in enhancing membrane stability and antioxidation [72,73], this result may indicat a recovery of membrane stability and a weakening of oxidative effects in the later periods of drought stress.

### Important KEGG pathways in drought stress response in sunflower

Through KEGG analysis, we identified several pathways that act simultaneously at multiple time points that have also been shown to play important roles in drought stress studies in sunflower and other plants, such as γ-aminobutyric acid (GABA), sucrose and starch metabolism, Terpene biosynthesis, and Photosynthesis and Carbon fixation.

GABA plays an important role in the response to abiotic stresses through ion channel regulation [74]. Exogenous addition of GABA can alleviate drought stress in sunflowers by increasing the levels of Pro, SS and total protein [75]. In our study, we found that the up-regulated genes were enriched on both day 7 and day 14 of drought stress, indicating the important role of endogenous GABA in drought response in sunflowers.

Plants mitigate the effects of drought stress by regulating the expression of carbohydrate-related genes [76], and sucrose and starch metabolism-related pathways have been identified in many drought studies, such as in *Aegilops tauschii* [77] and maize [78]. A previous study shown in sunflowers, genes related to sugar synthesis and starch degradation were up-regulated, whereas genes related to sucrose degradation are down-regulated under drought compared to control [22]. In this experiment, the "starch and sucrose metabolism" pathway was enriched by down-regulated genes at all 3 time points.

Carbon accumulated under drought conditions also promotes the synthesis of secondary metabolites, and terpene accumulation is involved in many functions in growth, development, and resistance to environmental stresses [79], and Terpenoid backbone biosynthesis pathway was found by Liang et al [23] in sunflower leaves. Our study also identified 2 terpen related pathways, Terpenoid backbone biosynthesis, Sesquiterpenoid, and triterpenoid biosynthesis, enriched by down-regulated genes.

Similar to the results of GO analysis, multiple photosynthesis-related pathways were enriched by down-regulated genes at day 14, Carbon fixation in photosynthetic organism pathway enriched by down-regulated DEGs in at least 2 time points simultaneously. These finding give additional evidence of the inhibition of photosynthesis at mid-period under drought stress.

### Major TFs involved in the drought tolerance

TFs as key regulators of transcription important in plant responses to drought stress. A large number of TFs were observed by iTAK in two inbred lines belonging to bHLH, AP2/ERF-ERF, MYB, NAC, and WRKY families.

The AP2/ERF TFs family is one of the largest plant-specific TFs families that share a well-conserved DNA-binding domain [80–82]. A lot of studies have shown genes belonging to AP2/ERF type TFs can increase tolerances to drought [83,84]. In this study, both the total number and up-regulation rate of genes belonging to the AP2/ERF TFs family increased with the extension of drought time, and DT was higher than DS (except DT21) at the same time point, which is in line with a previous study on tobacco [85]. This result indicates that sunflower AP/ERF family genes respond to drought stress mainly through up-regulated expression.

The proteins of MYC/MYB families are found in both plants and animals with varied functions [86,87]. Transgenic apple plants overexpressing Oryza sativa *OsMYB4* genes showed improved tolerance to drought stress by stimulating the expression of MYB TFs [88]. In this study, both iTak and Mapman analysis simultaneously found that the total number of DT was higher than that of DS on the 7th and 14th day of drought stress, and the proportion of up-regulated genes in DT was the highest on the 14th day of drought stress. Gene LOC110912287(MYB-like DNA-binding domain) has the highest up-regulated foldchange (1305 time).

WRKY proteins have been extensively studied for their important regulatory roles in plant defense and disease resistance [89]. WRKY3 [90] and WRKY93 [91] have been proved to enhance the drought tolerance of grape and wheat respectively. Li et al. identified 90 WRKY TFs in sunflower [92]. A previous study identified WRKY TFs enriched in the roots of a drought-tolerant sunflower variety (B71) [19], while another study found down-regulated expression of WRKY genes under drought stress. [22]. Our experiments found up-regulation of WRKY gene expression under drought stress, suggesting a positive regulatory role of WRKY genes in drought stress.

bHLH are widely distributed in all eukaryotes and are one of the largest families of TFs, playing a crucial role in plant growth, development, and stress response [93,94]. A previous study identified 183 bHLH genes in sunflower, of which *HabHLH159* and *HabHLH024* genes showed high expression under drought stress [95]. Another study on maize inbred lines also found that most bHLH genes were up-regulated under drought stress [78]. However, the opposite result was found in our study. In two sunflower self-incompatible lines material, the down-regulated expression of bHLH was dominant under drought stress, with 83.91% down-regulation at 14 day of DT.

A recent study by Li et al. [50] identified 150 NAC family genes at a genomic wide level in sunflower, of which 10 differentially expressed NAC genes were up-regulated and only 1 gene was down-regulated under drought stress, indicating that NAC genes play a positive regulatory role in drought. In contrast, another study showed that most NAC genes were down-regulated under drought stress [22]. Our results were consistent with the former, and both the number and the percentage of up-regulated NAC transcription factor genes were elevated with prolonged drought stress.

### Analysis of plant hormone signal transduction to drought stress

Phytohormones, including auxin, ABA, ethylene, JA, SA, Br, and gibberellins, play an important role in drought resistance by influencing the physiological processes of plants [96,97]. Many studies have shown that exogenous application of plant hormones can alleviate drought stress in sunflowers [98,99]. However, there are fewer reports on the drought stress response mechanisms of endogenous hormones in sunflowers. In this study, we analyzed the different responses of phytohormones in two sunflower varieties under drought stress via Mapman software.

ABA is one of the main plant hormones directly involved in drought stress response [100] and has been a popular research topic in crop drought tolerance [101]. ABA has a dual function in plant growth regulation [102], it promotes seedling development at low concentrations under well-watered conditions [101], while under stress conditions, it maintains plant survival by inhibiting stomatal opening and size increasing through massive accumulation [103]. Fernández C et al. found higher ABA content in DT varieties than DS varieties in sunflower under drought stress [104]. Sarazin Vivien suggests that drought tolerance is correlated with activating the ABA pathway, but not ABA overproduction [24]. Our experiments found more ABA-related pathway genes involved in response to drought stress, but could not demonstrate whether ABA content was elevated.

Ethylene induces stomatal closure through Ca^2+^ and NO signaling systems [105] to reduce water evaporation in response to drought stress. Meanwhile, ncreasing ethylene leads to leaf senescence and abscission, which to some extent affects yield [106–108]. Auxin is the dynamic plant hormone, that controls the growth and developmental processes by modulating the levels of auxin/indole acetic acid proteins [109]. A study identified synergistic efficiency of ethylene and Auxin in drought response in sunflower, the increased expression of ethylene-related genes promoted biosynthesis and basal transport of auxin to the root elongation zone, thereby inhibiting cell expansion in the root elongation zone [19]. Our results also found that more ethylene and auxin genes were expressed in DT under drought stress.

JA and its conjugates, such as methyl jasmonate (MeJA) and jasmonoyl-isoleucine (JA-Ile), collectively known as jasmonates (JAs). JAs are associated with the closure of stomata [110], and also plays role in modifying the root hydraulic conductivity under water-limited conditions to promote the uptake of water from the soil [111]. Salicylic acid (SA) is a natural endogenous phenolic compound, which plays a variety of regulatory roles in plant metabolism. There is a high rate of positive or negative crosstalk and interactions between JA and SA determining the ultimate response of plant to stress [112,113]. Our results found that JA and SA were reduced in DS, but some were elevated and some were reduced in DT, which was similar to Fernandez et al [104], who found that SA levels under drought conditions were decreased in the DS compared to the control conditions, whereas JA levels changed under drought conditions, the direction of change was not consistent within the DT and DS.

### Search for key modules and genes through WGCNA analysis

WGCNA is a progressive analysis method in which variable genes are divided into co-expression modules through an unsigned network based on DEGs [25]. Each module was correlated with various traits. In this study, we found a correlation between 2 modules and 6 different traits. The blue 4 module was positively related to LSA, while the lightslateblue module was positively related to REC, POD, Pro, with correlation coefficients of more than 0.8.

Pathway analysis showed genes in blue 4 module enriched in glycerolipid metabolism and Glycerophospholipid metabolism, two in the top ten genes ranked by cytoHubba plug-in were annotated Monogalactosyldiacylglycerol (MGDG) synthase. Glycerolipids, including phospholipids (PLs) and glycolipids (GLs), are the most abundant lipids in plants and are involved in responses and tolerance to abiotic conditions such as drought, heat, and low temperature [114,115]. Chmielewska et al. observed many glycerophospholipids strongly accumulated under drought stress in hulled barley [116]. Several glycerophospholipids were found highly accumulated in ryegrass in responses to drought stress [117]. Our results suggest that glycerophospholipid metabolism also plays an important role in sunflower drought response, glycerol metabolism-related genes may affect LSA in certain ways.

The maintenance of inorganic phosphate homeostasis is essential for plant growth and yield [118]. GO analysis showed “cellular response to phosphate starvation” was the GO-terms with the most genes and lowest p-value. Phosphate starvation response precedes drought response in field-grown plants according to Nagatoshi Y research, Mild drought reduces inorganic phosphate levels in the leaves to activate the phosphate starvation response (PSR) in field-grown soybean plants [119]. López C M et al. put forward that besides ABA-mediated reaction, the acquisition of phosphate is also very important to the drought resistance of this common bean genotype [120]. It can be seen that phosphorus supplementation can cope with early drought stress.

The PK family involves many signaling pathways that are triggered during stress and development processes [121]. Among them, Receptor-like kinases (RLKs) are transmembrane proteins with an N-terminal extracellular domain and a C-terminal kinase domain that play essential roles in responses to environmental stresses [122]. Ouyang S Q et al. found overexpression of RLK gene OsSIK1 in rice increases plant tolerance through suppressed stomatal development and decreased stomatal density in leaves, which promoted drought tolerance by diminishing water loss [123]. LRK2 genes enhance drought tolerance in plants by increasing the number of lateral roots compared with the wild types at the vegetative stage [124]. In this study, we found two top-ranked RLK genes (MSTRG. 22454, LOC110885040) in blue 4 module, which may have an important impact on stomata and lateral root number.

Although genes in lightslateblue module were far more than in blue 4, but only two KEGG pathways were enriched, and the “Ribosome biogenesis in eukaryotes” pathway was the most significant. CytoHubba analysis showed that the top 10 genes all encoded ribosomal proteins. Up-regulated genes were enriched in the KEGG pathway of ‘ribosome biogenesis in eukaryotes’ in wheat(*Triticum aestivum L*.) leaves induced by H_2_S under drought stress according to H Li et al [125]. But DEGs involved in the ribosome, ribosome biogenesis were all down-regulated in drought-tolerant Eruca under polyethylene glycol-simulated drought stress. Genes enriched in “Ribosome biogenesis in eukaryotes” were down-regulated in Carlic according to Xiangjun Zhou [126], but the mechanism of ribosomal proteins in a drought stress still needs further work studied.

## Supporting information

S1 Fig Volcano map for DEGs. (TIF)

S2 Fig GO analysis results of DEGs. (TIF)

S3 Fig KEGG dot plots of DEGs. (TIF)

S4 Fig Mapman analysis of DEGs at 7 day of drought stress. (TIF)

S5 Fig Mapman analysis of DEGs at 21 day of drought stress. (TIF)

S6 Fig Clustering dendrogram of samples based on their Euclidean distance to detect outliers. (TIF)

S7 Fig Analysis of network topology for various soft-thresholding powers. (TIF)

S1 Table Primers used in RT-qPCR. (XLSX)

S2 Table Physiological traits of twenty-four samples. (XLSX)

S3 Table DEGs detected in DS and DT. (XLSX)

S4 Table Results of Gene Ontology Analysis. (XLSX)

S5 Table Common GO-terms across DS and DT at same drought stress time points. (XLSX)

S6 Table Results of KEGG analysis. (XLSX)

S7 Table Common KEGG pathways across DS and DT at same drought stress time points. (XLSX)

S8 Table Transcription factors identified by iTAK. (XLSX)

S9 Table GO analysis of genes in two modules after redundancy by Revigo. (XLSX)

S10 Table KEGG analysis of genes in two modules. (XLSX)
