# Supplementary figures and images for: Time-course transcriptome and WGCNA analysis revealed the drought response mechanism of two sunflower inbred lines
Source: PLoS One. 2022 Apr 1;17(4):e0265447. doi: 10.1371/journal.pone.0265447 (PMC8974994; doi:10.1371/journal.pone.0265447)

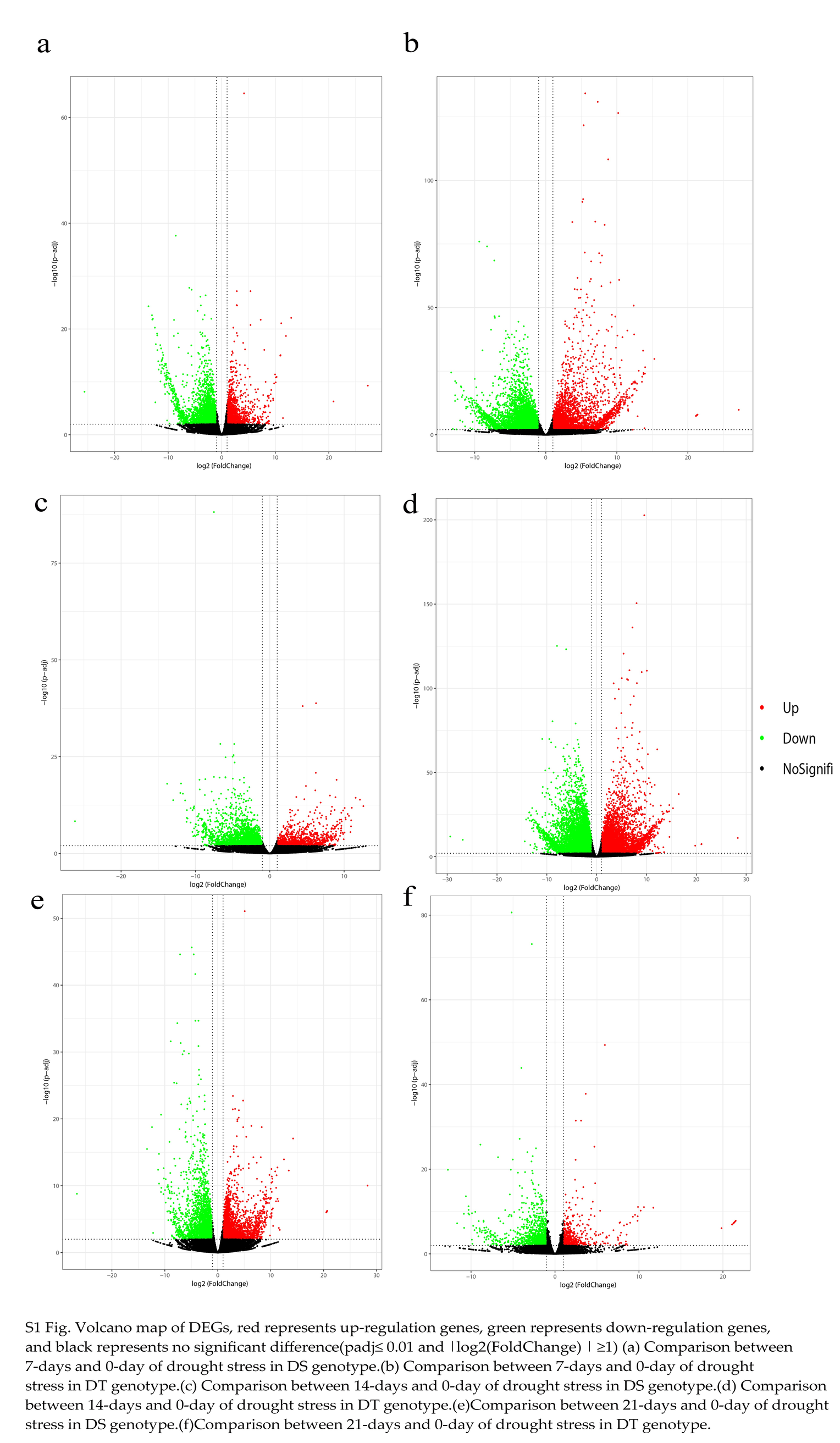

Supplement: S1 Fig — (TIF) [file pone.0265447.s001.tif]

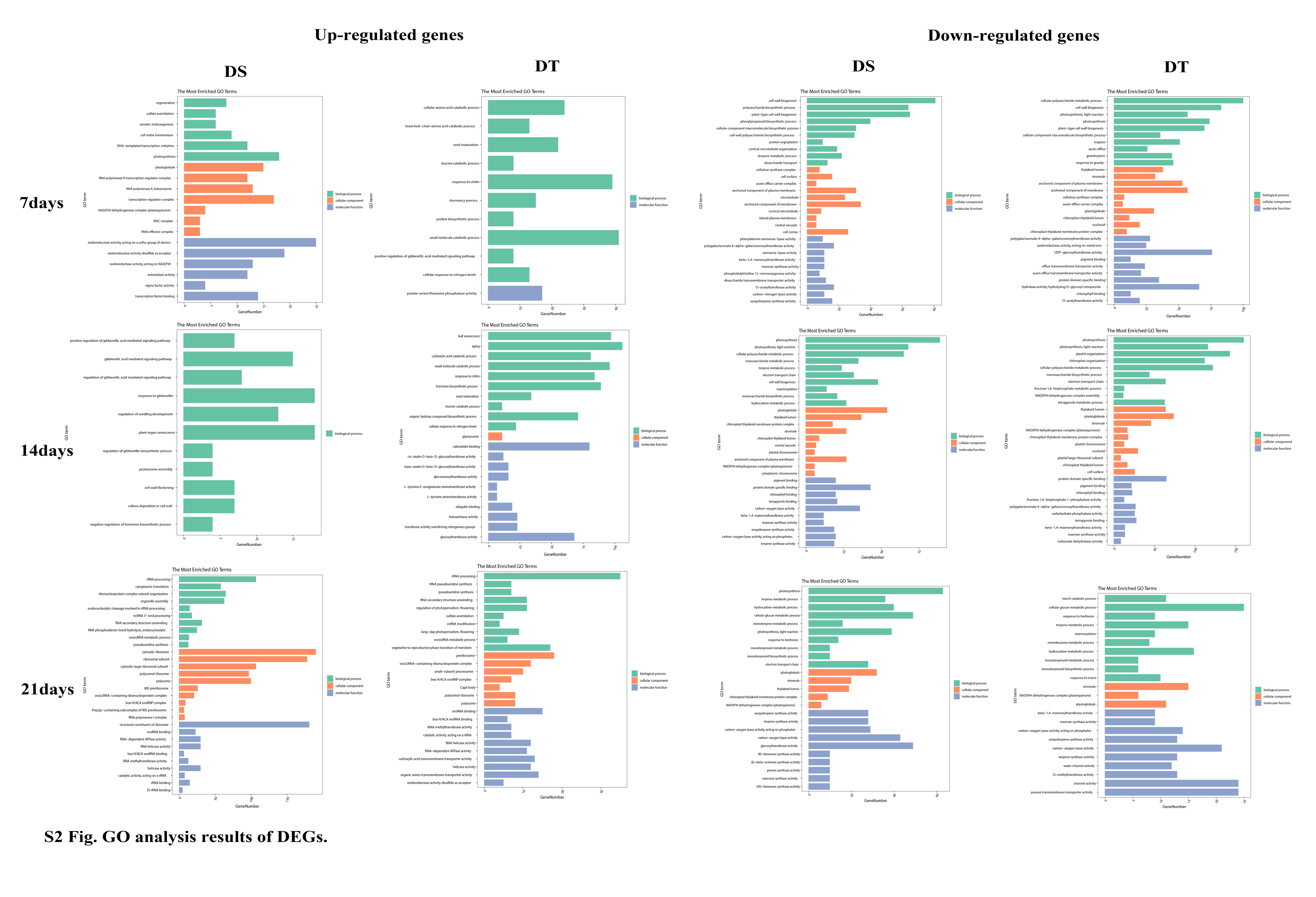

Supplement: S2 Fig — (TIF) [file pone.0265447.s002.tif]

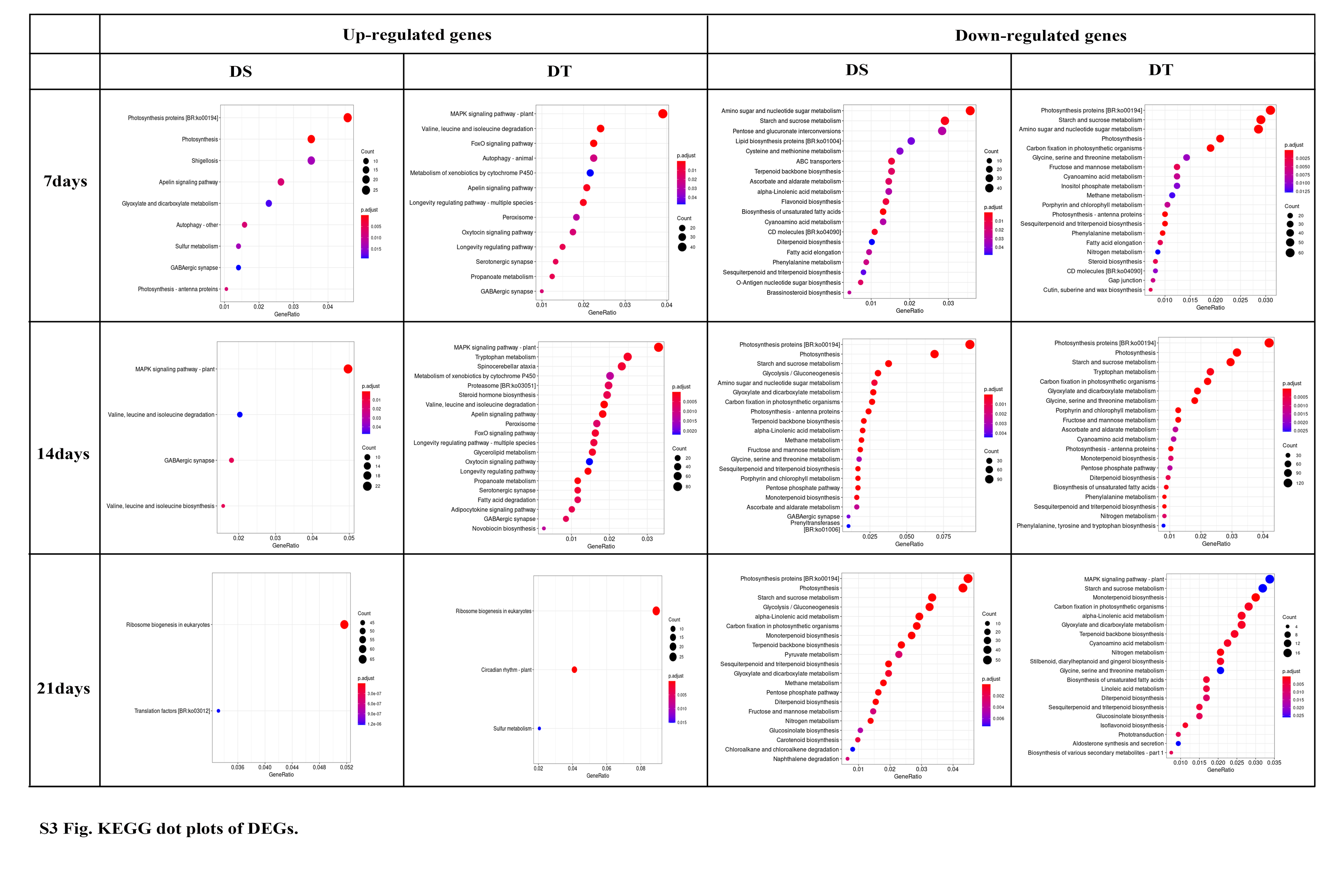

Supplement: S3 Fig — (TIF) [file pone.0265447.s003.tif]

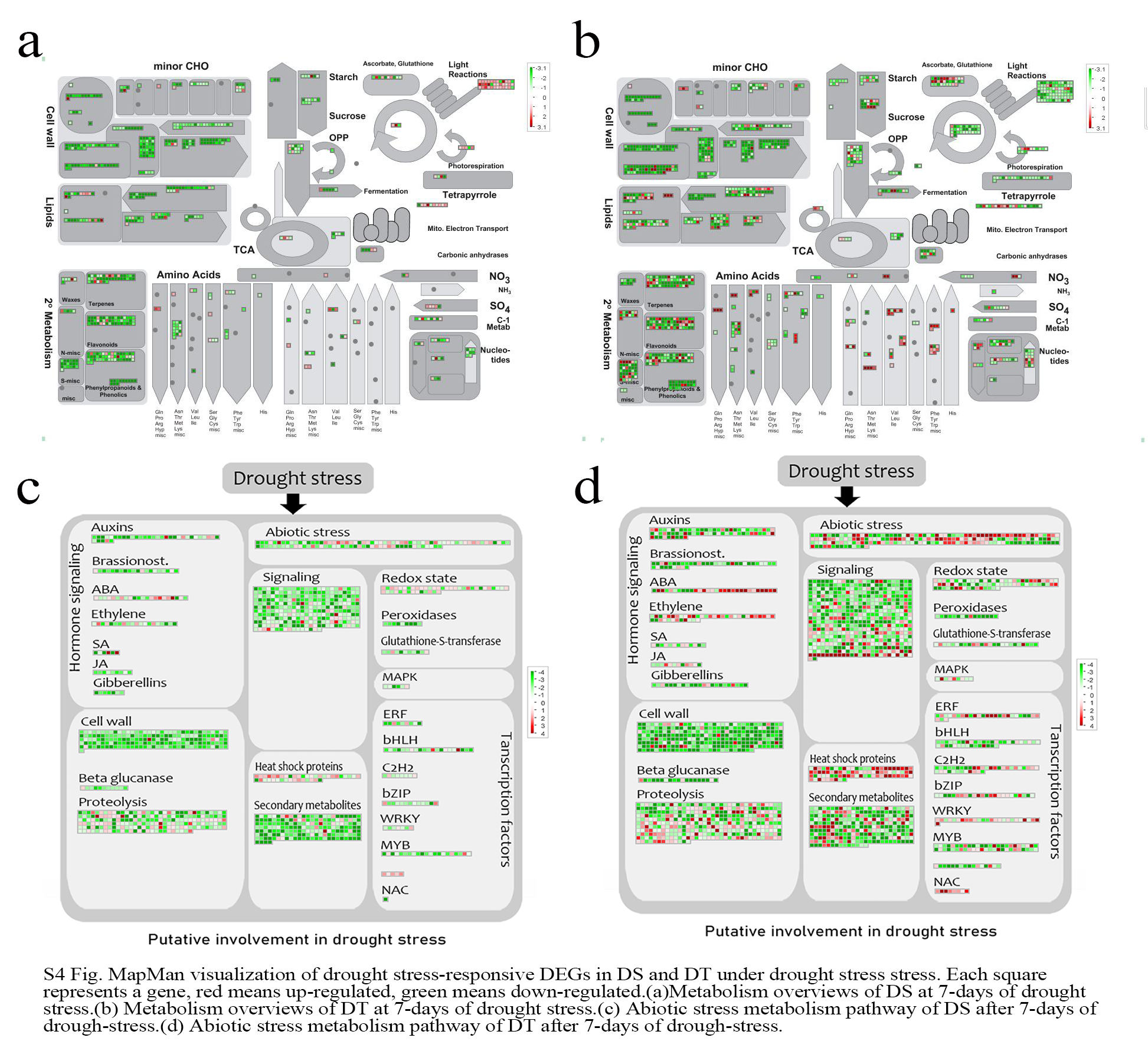

Supplement: S4 Fig — (TIF) [file pone.0265447.s004.tif]

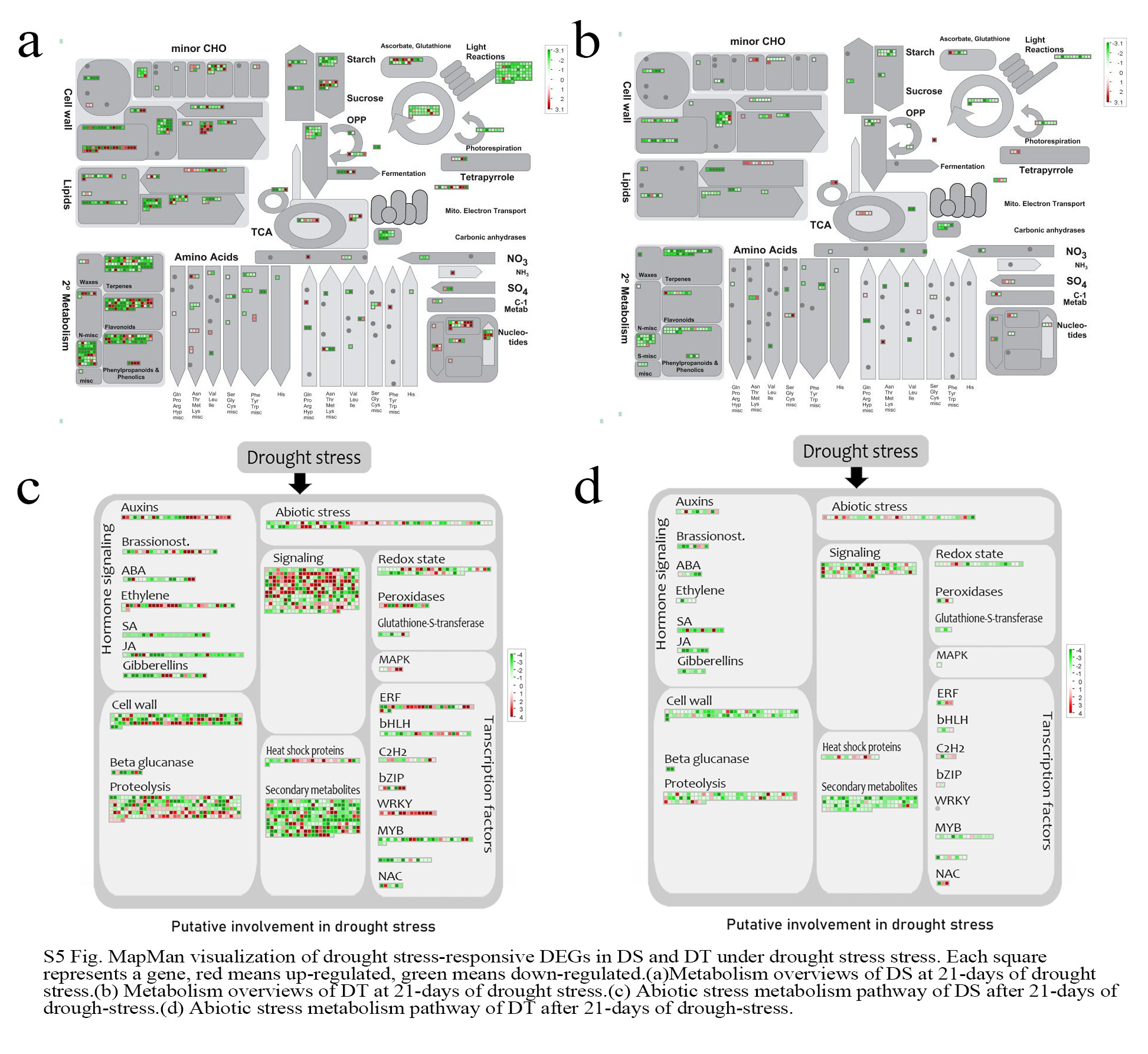

Supplement: S5 Fig — (TIF) [file pone.0265447.s005.tif]

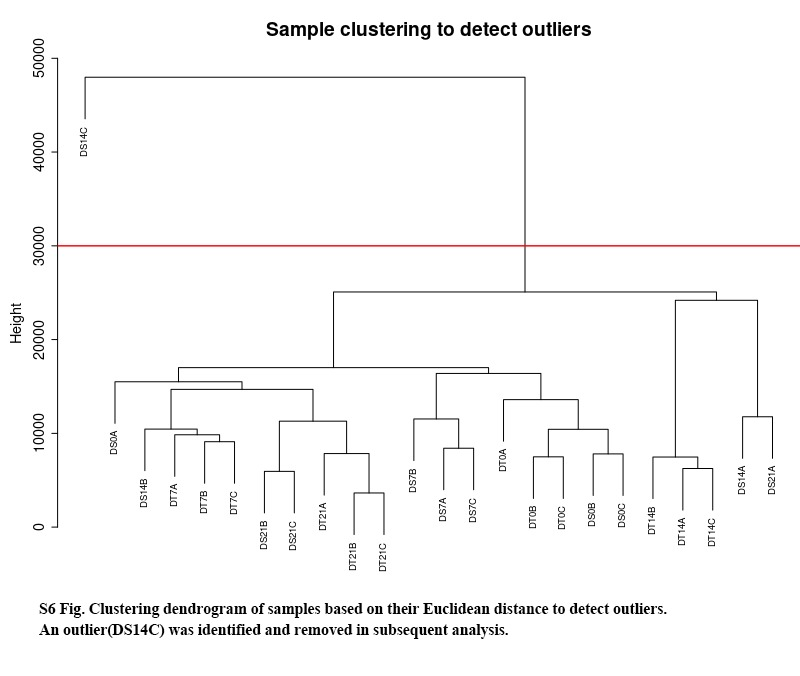

Supplement: S6 Fig — (TIF) [file pone.0265447.s006.tif]

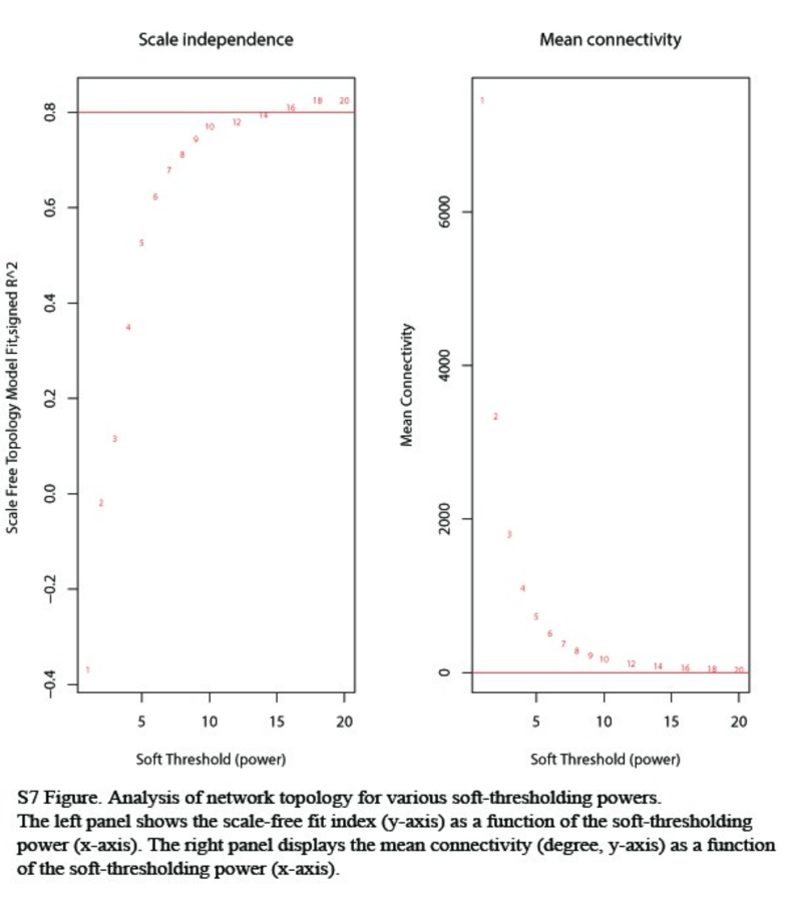

Supplement: S7 Fig — (TIF) [file pone.0265447.s007.tif]
